# Two (methyl­sulfan­yl)benzyl-derivatized palladium–N-heterocyclic carbene complexes – same formula type but not isotypic

**DOI:** 10.1107/S2056989026003385

**Published:** 2026-04-10

**Authors:** Matthias Weil, Laura Ielo, Prasad M. Kathe, Katharina Bica-Schröder

**Affiliations:** a Institute for Chemical Technologies and Analytics, Division of Applied Solid State Chemistry, TU Wein, Getreidemarkt 9/E164-05-1, 1060 Vienna, Austria; b Institute of Applied Synthetic Chemistry, TU Wien, Getreidemarkt 9/E163-03-5, 1060 Vienna, Austria; Universität Greifswald, Germany

**Keywords:** crystal structure, N-heterocyclic carbene (NHC), NHC–palladium complex, square-planar coordination, symmetry relationships

## Abstract

Although the two title palladium–N-heterocyclic carbene (Pd–NHC) complexes, namely, di­chlorido­{1-methyl-3-[2-(methyl­sulfan­yl)benz­yl]-2*H*-imidazol-2-yl­idene-κ*C*^2^}(pyridine-κ*N*)palladium(II) and di­bromido­{1-methyl-3-[2-(methyl­sulfan­yl)benz­yl]-2*H*-imidazol-2-yl­idene-κ*C*^2^}(pyridine-κ*N*)palladium(II), have the same formula type [Pd*X*_2_(C_5_H_5_N)(C_12_H_14_N_2_S)] (*X* = Cl and Br), and the conformations of the corresponding mol­ecules are very similar, they crystallize in different space-group types: the PdCl_2_ complex in *P*1 with *Z* = 4 and two mol­ecules in the asymmetric unit, and the PdBr_2_ complex in *C*2/*c* with *Z* = 8 and one mol­ecule in the asymmetric unit. The symmetry relationship between the two crystal structures is of *translationengleiche* type with index 2 (t2). In both mol­ecular structures, the central palladium(II) atom has a slightly distorted square-planar coordination environment with the C- and N-bound organic ligands in a *trans* arrangement. In the crystals, weak C—H⋯*X* inter­actions lead to the formation of supra­molecular layers parallel to (010) for the PdCl_2_ complex and (100) for the PdBr_2_ complex.

## Abstract 2

The two palladium–N-heterocyclic carbene (Pd–NHC) title complexes have the same formula type but crystallize in different space-group types, *viz*. the PdCl_2_ complex in *P*1 with *Z* = 4 and two mol­ecules in the asymmetric unit, and the PdBr_2_ complex in *C*2/*c* with *Z* = 8 and one mol­ecule in the asymmetric unit.

## 1. Chemical context

Palladium–N-heterocyclic carbene (Pd–NHC) complexes have emerged as a prominent class of organometallic compounds due to their exceptional stability, tunable electronic properties, and versatile reactivity. Since the pioneering isolation of stable NHCs in the early 1990s (Arduengo *et al.*, 1991 ▸), these ligands have become central to modern coordination chemistry and homogeneous catalysis. The strong *σ*-donating nature of NHCs generates highly electron-rich palladium centres, which lowers activation barriers for oxidative addition and enables the activation of challenging substrates such as aryl chlorides and sterically hindered electrophiles. In addition, the robust Pd–C(NHC) bond imparts superior thermal and chemical stability relative to phosphine-based systems, resulting in longer catalyst lifetimes, reduced ligand dissociation, and improved reproducibility under catalytic conditions (Kantchev *et al.*, 2007 ▸). As a result of these favourable properties, Pd–NHC complexes have been widely applied as highly efficient precatalysts in carbon–carbon and carbon–heteroatom bond-forming reactions, including Suzuki–Miyaura, Heck, Sonogashira, and Buchwald–Hartwig couplings (Çekirdek *et al.*, 2014 ▸). Beyond classical cross-coupling, Pd–NHC systems have also demonstrated high activity in allylic substitution (Bai *et al.*, 2016 ▸), carbonyl­ative couplings, cyclo­propanation, and multicomponent reactions, highlighting their versatility and mechanistic flexibility (Fortman & Nolan, 2011 ▸).

Among the various NHC ligand families, imidazolium-based NHCs represent the most extensively studied and widely employed class. Imidazolium salts are readily accessible, structurally versatile, and serve as convenient precursors for *in situ* or isolated carbene generation. Palladium complexes derived from imidazolium-based NHCs often display an optimal balance between σ-donor strength and steric tunability, contributing to their high catalytic efficiency and operational robustness. Substitution at the N-positions of the imidazolium ring enables systematic modulation of steric bulk and electronic properties, which has been exploited to improve activity, selectivity, and resistance to catalyst deactivation (Kantchev *et al.*, 2007 ▸).

Imidazolium-derived Pd–NHC complexes have also proven to be particularly effective in supported and heterogeneous catalyst designs, where strong metal–ligand inter­actions help suppress palladium aggregation and leaching. Polymer-anchored and resin-supported imidazolium-based Pd–NHC systems exhibit excellent recyclability and stability while maintaining high catalytic activity, making them attractive for sustainable and industrially relevant processes (Yue *et al.*, 2021 ▸). Consequently, imidazolium-based Pd–NHC complexes continue to serve as a cornerstone in the development of next-generation palladium catalysts for both homogeneous and heterogeneous applications.

In the context given above, we report here the syntheses, characterization and crystal structure determinations of two imidazolium-derived Pd–NHC compounds, [Pd*X*_2_(C_5_H_5_N)(C_12_H_14_N_2_S)] (*X* = Cl, Br), which contain 2-(methyl­sulfan­yl)benzyl and methyl moieties at the 1 and 3 positions of the imidazolium ring, and a pyridine ligand next to the two halogen atoms.
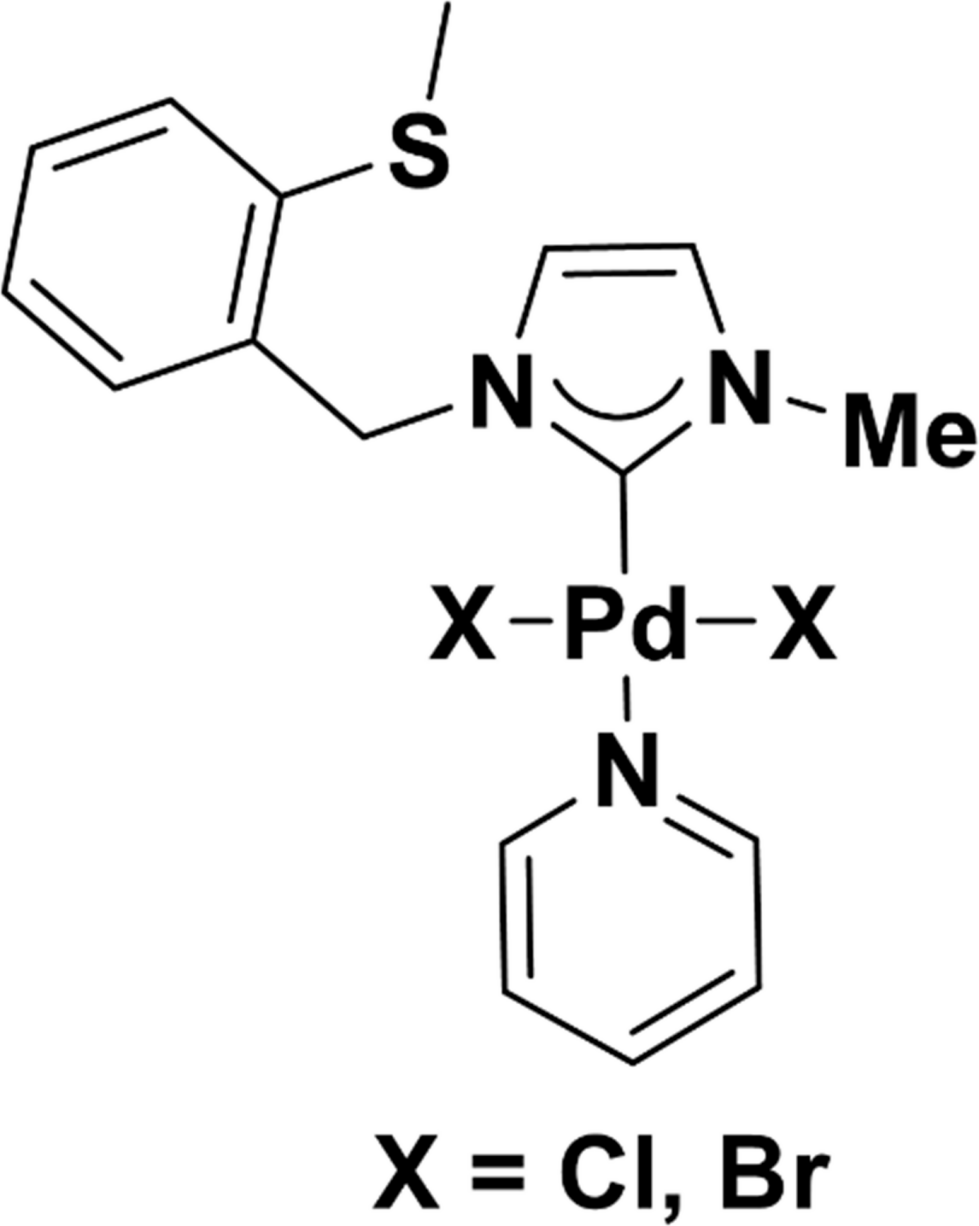


## 2. Structural commentary

The two title compounds **7a** and **7b** have the same formula type and differ only in terms of their halogen atoms (*X* = Cl for **7a** and Br for **7b**). Structurally, one might therefore expect an isotypic relationship, but this is not the case: **7a** and **7b** crystallize in different space-group types, *viz*. **7a** in *P*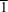 with *Z* = 4 and two mol­ecules in the asymmetric unit (denoted with suffixes *A* and *B* for corresponding atoms), and **7b** in *C*2/*c* with *Z* = 8 and one mol­ecule in the asymmetric unit. The *C*-centred monoclinic cell can be related to the primitive triclinic cell by the transformation matrix **–b**,**1/2a+1/2b**,**c** (the triclinic cell actually represents the reduced monoclinic cell). Space group *P*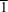 is a *translationengleiche* maximal subgroup of *C*2/*c*, and the symmetry relationship (Bärnighausen, 1980 ▸; Müller & de la Flor, 2024 ▸) between the two space groups is given as *C*2/*c*—t2→ *P*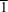.

**Figure 1:**
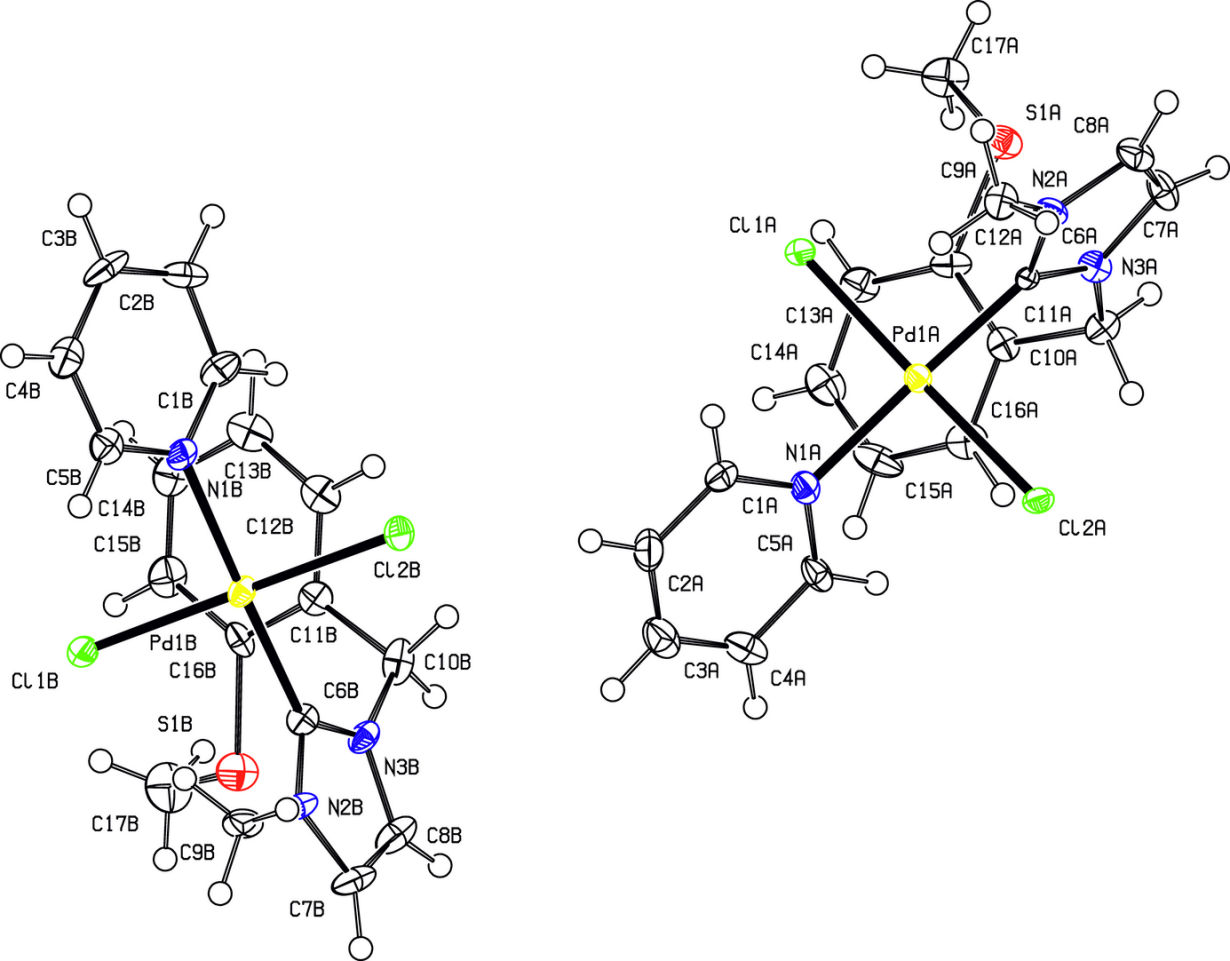
Mol­ecular structures of the two mol­ecules present in the asymmetric unit of **7a**. Displacement ellipsoids are drawn at the 50% probability level; H atoms are given as spheres of arbitrary size.

The coordination environment of the palladium(II) atoms in the two compounds shows the characteristic square-planar environment (Figs. 1 ▸ and 2 ▸). In both mol­ecular structures, the bond lengths to the carbene atom (C6) and to the *trans*-positioned pyridine N atom (N1) are very similar, with the Pd—C bond lengths being approximately 0.1 A shorter than the Pd—N bond lengths (Tables 1 ▸ and 2 ▸). Only the Pd—*X* bond lengths differ significantly due to the different radii of the halogen atoms. The *τ*_4_ descriptor (Yang *et al.*, 2007 ▸) deviates slightly for the Pd atoms from the ideal value of 0 for an ideal square-planar coordination and amounts to 0.03 for both Pd atoms in **7a** and is marginally greater with 0.05 for **7b**. This deviation is also seen in the angular distortions with numerical values compiled in Table 1 ▸ (**7a**) and 2 (**7b**). In **7a**, dihedral angles between the pyridine ring and the imidazolium ring, between the Pd*X*_2_CN coordination plane and the imidazolium ring, and between the Pd*X*_2_CN coordination plane and the pyridine ring are 26.0 (3), 71.0 (3) and 45.3 (2)° for mol­ecule *A*, and 18.6 (3), 70.4 (3) and 52.3 (2)° for mol­ecule *B.* In **7b**, the corresponding dihedral angles are 23.3 (2), 74.45 (17) and 51.47 (14)°. The benzyl unit is perpendicular to the imidazolium ring, with dihedral angles between the least-squares planes of these units of 89.1 (3)° for mol­ecule *A* and 89.4 (4)° for mol­ecule *B* in **7a**, and of 89.91 (1)° in **7b**. Other bond lengths and angles of the organic moieties are within normal ranges.

**Figure 3:**
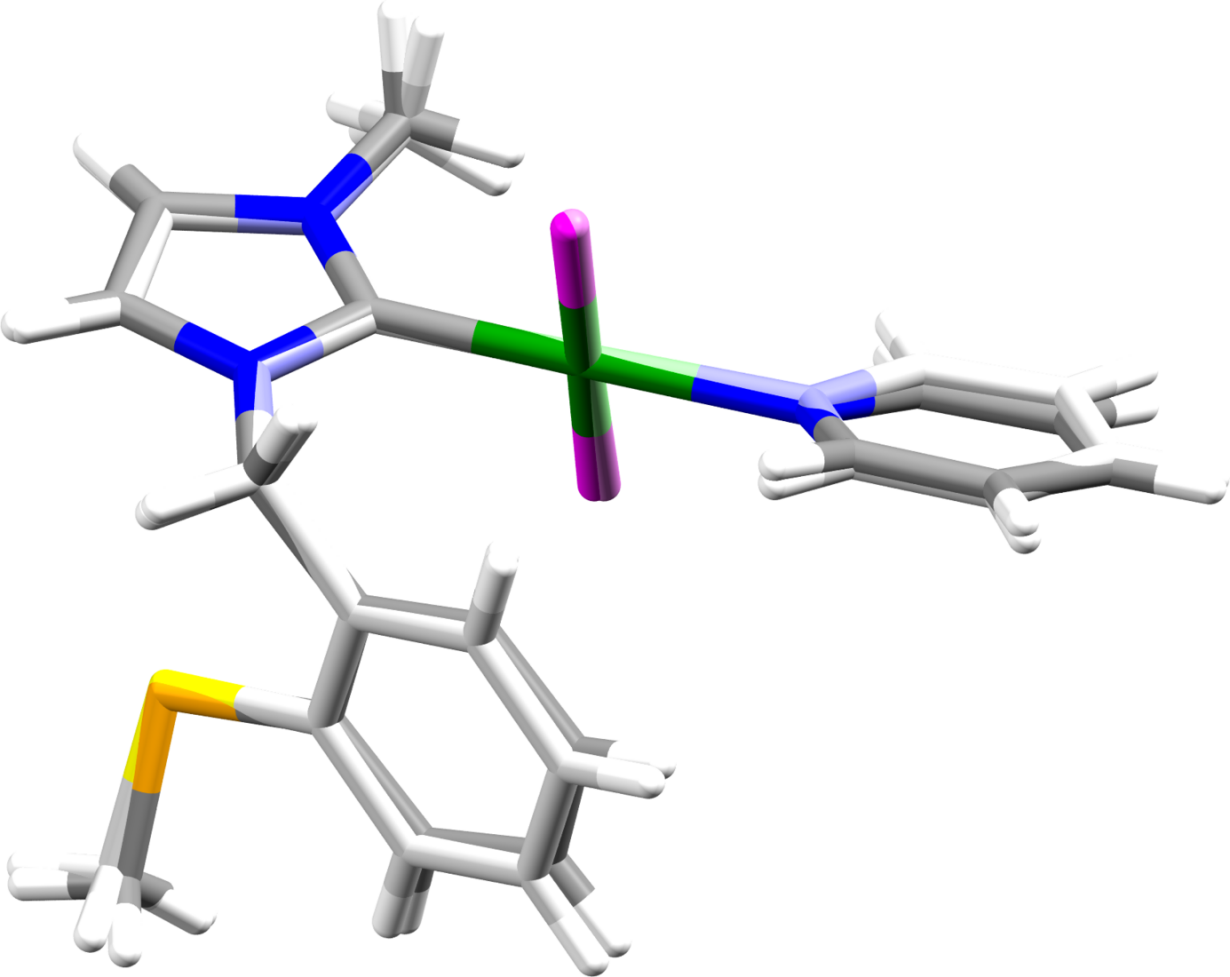
Overlay plot of the two independent mol­ecules present in **7a**. Mol­ecule *B* is shown in lighter colours.

The two independent mol­ecules in **7a** exhibit a similar conformation (root-mean-square deviation 0.1054 Å, max. deviation 0.2236 Å), as can be seen in an overlay plot (Fig. 3 ▸). Corresponding data for the superimposition of the two mol­ecules of **7a** with the mol­ecule of **7b** show comparable values (0.1046, 0.2787 Å for mol­ecule *A*; 0.1216, 0.2566 Å for mol­ecule *B*) and thus confirm the great similarity of the mol­ecular structures in the two compounds.

## 3. Supra­molecular features

**Figure 4:**
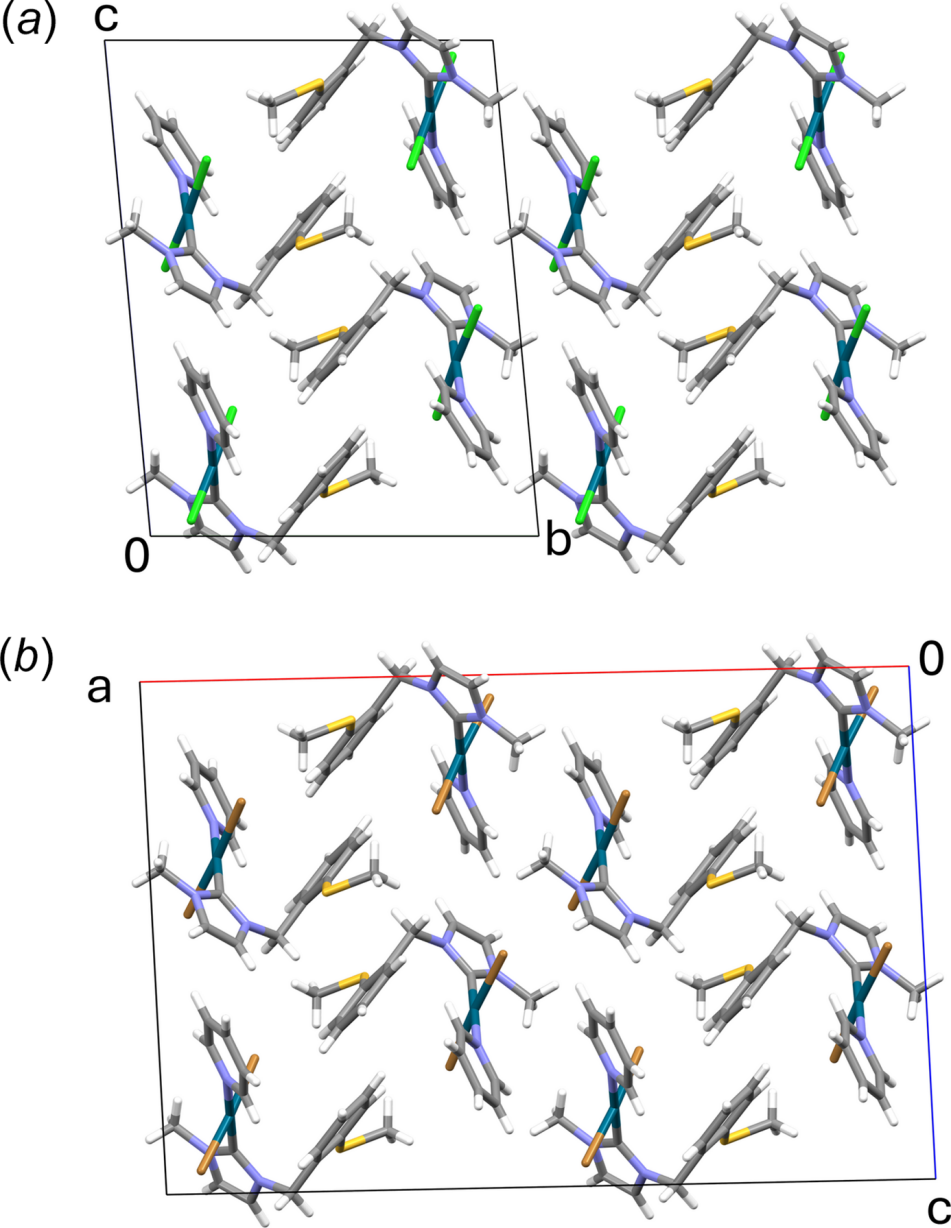
Crystal packing in the structures of **7a** in a view along [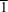
00] (*a*) and of **7b** in a view along [010] (*b*).

**Figure 5:**
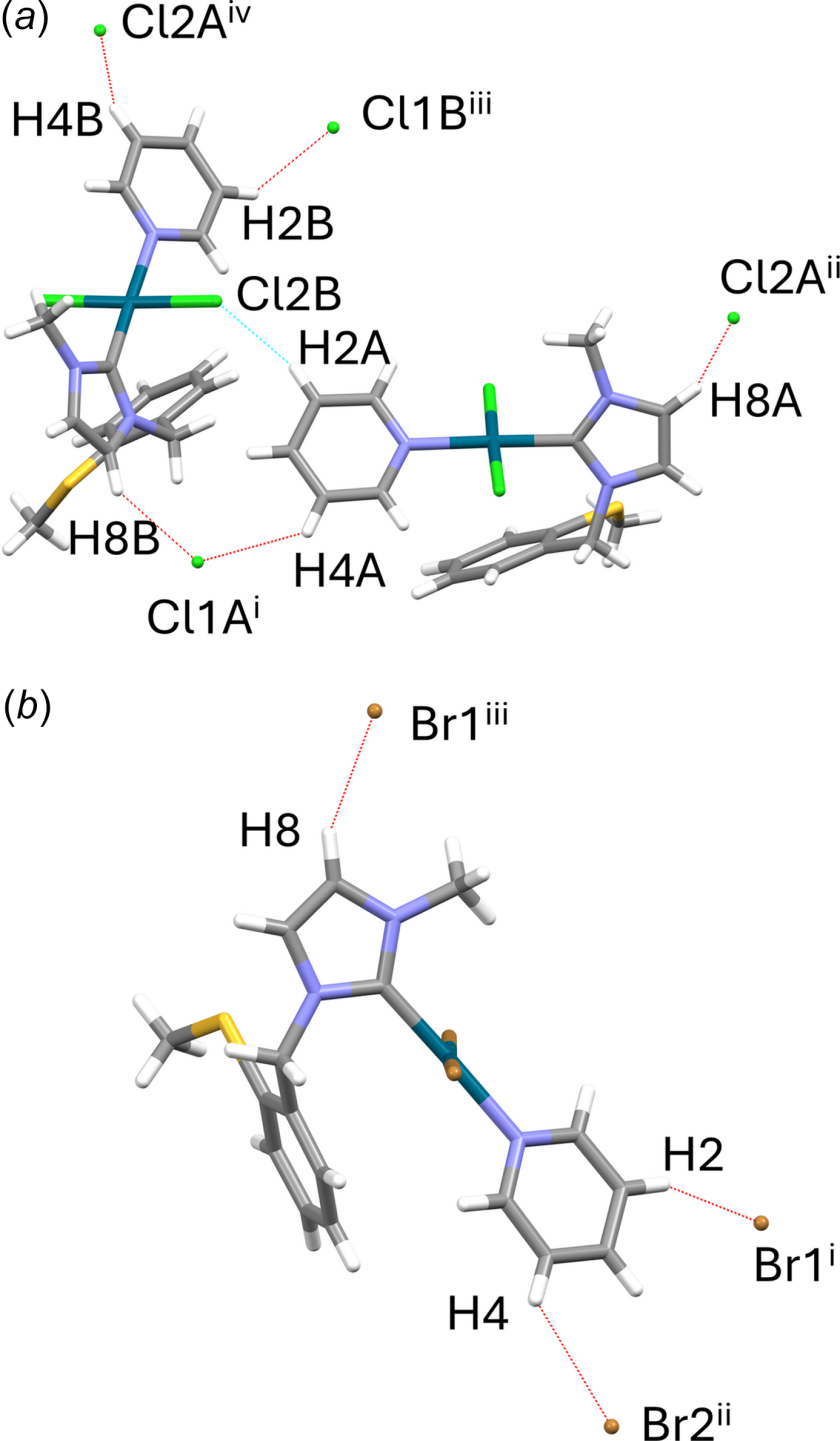
C—H⋯*X* inter­actions (dashed lines) in the crystal structures of **7a** (*a*) and **7b** (*b*). For clarity, only inter­actions in which a mol­ecule with its donor groups is visible are shown here. Symmetry codes refer to Tables 3 ▸ and 4 ▸, respectively.

The close relationship of **7a** and **7b** is also reflected in the packing of the mol­ecules in the two individual crystal structures, as Fig. 4 ▸ clearly illustrates. In both crystal structures, weak inter­molecular C—H⋯*X* inter­actions involving aromatic C—H groups of the imidazolium or pyridine rings as donors are present (Tables 3 ▸ and 4 ▸; Fig. 5 ▸). The supra­molecular layers formed in this way are flanked on both sides by the (methyl­sulfan­yl)benzyl side arms. For **7a**, these layers extend parallel to (010), and for **7b** parallel to (100).

No noticeable π–π stacking can be observed in either structure. Weak inter­actions between the ring centres of gravity (*Cg*) of aromatic rings and methyl (for **7a**) or methyl­ene (for **7b**) H atoms (Tables 3 ▸ and 4 ▸) might consolidate the packing in the two crystal structures.

## 4. Database survey

Searches of the Cambridge Structure Database (CSD, version 25.3.1; Groom *et al.*, 2016 ▸) were performed with the ConQuest routine (Bruno *et al.*, 2002 ▸) using a Pd*X*_2_ (*X* = Cl, Br) group N-bonded to pyridine and C-bonded to imidazolium moieties. This resulted in about 200 hits for *X* = Cl, and 60 for *X* = Br. In all these structures, the square-planar coordination of the central palladium(II) atom with a *trans* disposition of the organic ligands is retained. In order to better tailor the database search to the title compounds, additional sulfur atoms were considered, which significantly reduced the number of hits. Of the eleven structures obtained, only five contain sulfur in the form of comparable thio­ethers, *i.e*. with the S atoms bound to two neighbouring C atoms. In FOXLUD (Pasyukov *et al.*, 2023 ▸), MOHPOP (Lohre *et al.*, 2008 ▸) and QAVYIZ (Pasyukov *et al.*, 2022 ▸), the S atom is directly bound to one of the C atoms of the imidazolium ring, whereas in GOWBIH (Shevchenko *et al.*, 2024 ▸) the S atom is part of a (phenyl­sulfan­yl)methyl moiety bound to one of the C atoms of the imidazolium ring and in SIKGOL01 (Karthik & Gandhi, 2018 ▸) of a dibenzo­thio­phene moiety bound to one of the N atoms of the imidazolium ring.

## 5. Synthesis and crystallization

**Figure 6:**
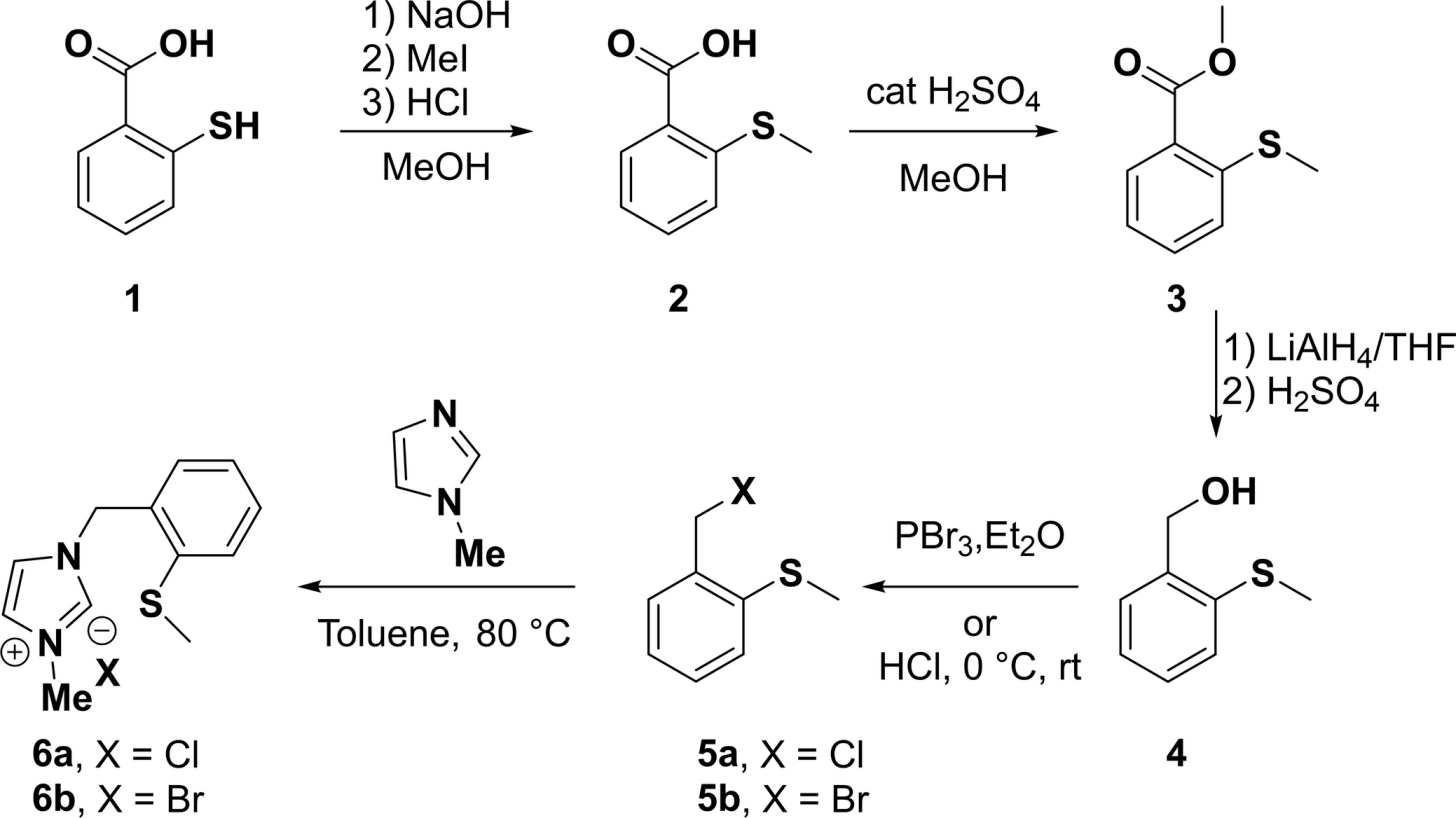
Synthesis scheme to obtain precursor compounds **2**–**6a**,**b**.

The synthesis of compounds **2**–**6a**,**b** was carried out according to literature procedures (Huynh *et al.*, 2010 ▸) and is schematically shown in Fig. 6 ▸.

*Synthesis of 1-methyl-3-[2-(methyl­sulfan­yl)benz­yl]-1H-imidazol-3-ium chloride* (**6a**). 1-(Chloro­meth­yl)-2-(methyl­sulfan­yl) benzene (1.15 mmol) was added to a solution of 1-methyl­imidazole (1.15 mmol) in toluene. The reaction mixture was stirred for 24 h at 353 K and after that the solvent was removed *in vacuo*. The white residue was washed with toluene and Et_2_O in order to obtain a white powder in 78% yield (229 mg).

*Synthesis of 1-methyl-3-[2-(methyl­sulfan­yl)benz­yl]-1H-imidazol-3-ium bromide* (**6b**). 1-(Bromo­meth­yl)-2-(methyl­sulfan­yl)benzene (2.7 mmol) was added to a solution of 1-methyl­imidazole (2.7 mmol) in toluene. The reaction mixture was stirred for 24 h at 353 K and after that the solvent was removed *in vacuo*. The white residue was washed with toluene and Et_2_O in order to obtain a white powder in 87% yield (707 mg).

**Figure 7:**
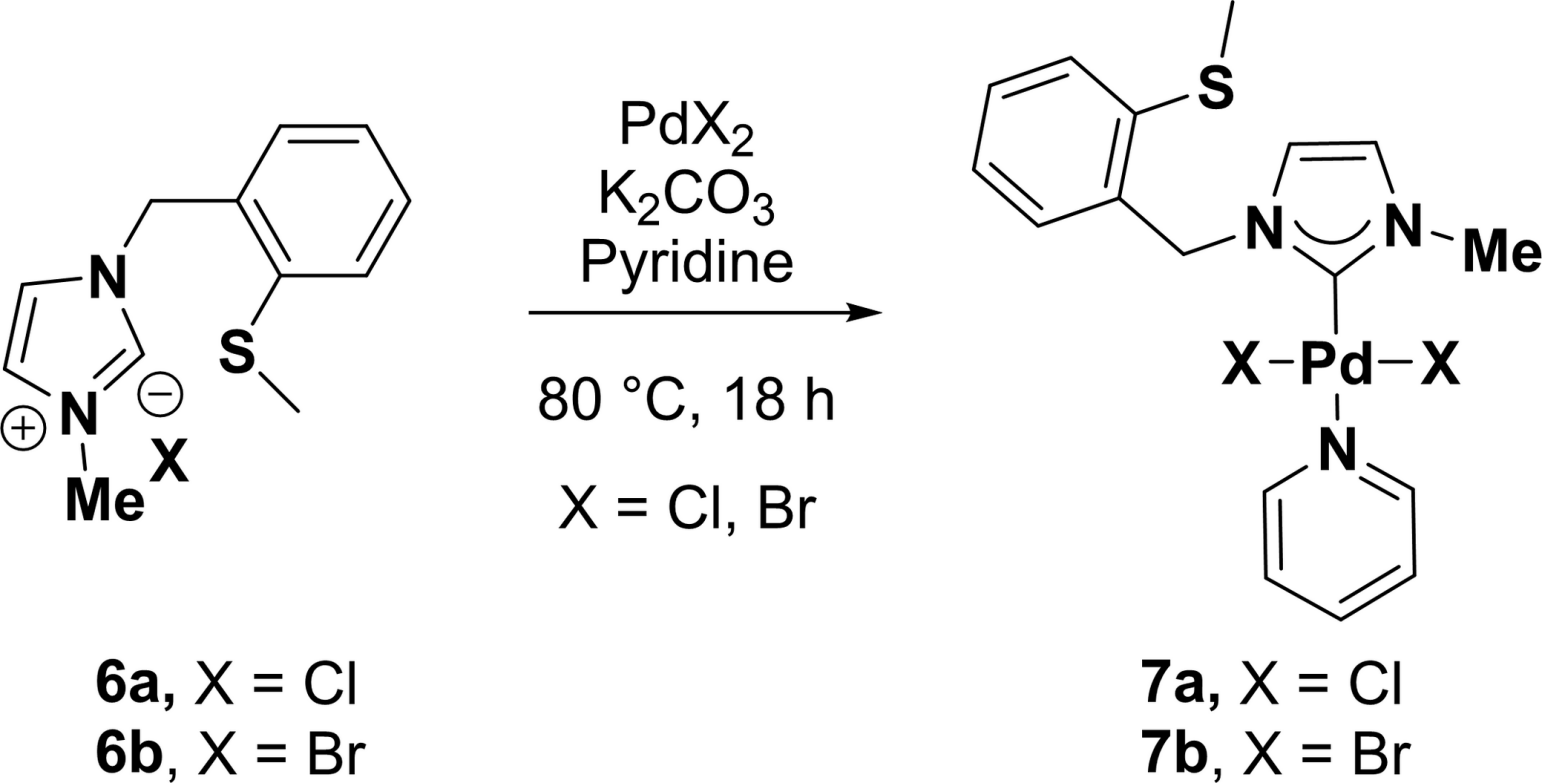
Synthesis scheme to obtain the title compounds **7a**,**b**.

The synthesis of compounds **7a**,**b** was carried out according to literature procedures (O’Brien *et al.*, 2006 ▸) and is schematically shown in Fig. 7 ▸.

*Synthesis of 1-methyl-3-[2-(methyl­sulfan­yl)benz­yl]-1H-imidazol-3-ium chloride Pd(NHC) complex* (**7a**). A vial was charged with PdCl_2_ (0.19 mmol), **6a** (0.2 mmol), K_2_CO_3_ (0.75 mmol) and a stir bar. Pyridine (1 ml) was added, the vial was capped with a Teflon®-lined screw cap and heated under vigorous stirring for 16 h at 353 K. After cooling to room temperature, the reaction mixture was diluted with DCM and passed through a short pad of silica gel covered with a pad of Celite eluting with DCM until the product was completely recovered. DCM was removed *in vacuo*. The pure complex **7a** was isolated after silica column chromatography (DCM 100%) as yellow crystals in 49% yield (50 mg).

*Synthesis of 1-methyl-3-[2-(methyl­sulfan­yl)benz­yl]-1H-imidazol-3-ium bromide Pd(NHC) complex* (**7b**). A vial was charged with PdBr_2_ (0.15 mmol), **6b** (0.16 mmol), K_2_CO_3_ (0.75 mmol) and a stir bar. Pyridine (0.75 ml) was added, the vial was capped with a Teflon®-lined screw cap and heated under vigorous stirring for 16 h at 373 K. After cooling to room temperature, the reaction mixture was diluted with DCM and passed through a short pad of silica gel covered with a pad of Celite eluting with DCM until the product was completely recovered. DCM was removed *in vacuo*. The pure complex **7b** was isolated after silica column chromatography (100% DCM) as orange crystals in 68% yield (60 mg).

^1^H and ^13^C NMR spectra of **6a**,**b** and **7a**,**b** are available as electronic supplementary information (ESI).

## 6. Refinement

Crystal data, data collection and structure refinement details are summarized in Table 5 ▸. For the refinement of both crystal structures, hydrogen atoms were placed geometrically and refined with a riding model. Their *U*_iso_(H) values were constrained to 1.5 × *U*_eq_ of the parent carbon atoms for the methyl groups and to 1.2 × *U*_eq_ for all other C-bound hydrogen atoms. The crystal of **7a** consisted of two domains that are related by a 180° rotation about [100]. Intensity data were finally processed in the HKLF5 format, revealing a refined ratio of the two domains of 0.56:0.44. One reflection (001) was obstructed from the beam stop and was omitted from refinement. For **7b**, likewise one reflection (200) was omitted due to obstruction from the beam stop.

## Supplementary Material

Crystal structure: contains datablock(s) 7b, 7a. DOI: 10.1107/S2056989026003385/yz2077sup1.cif

Structure factors: contains datablock(s) 7b. DOI: 10.1107/S2056989026003385/yz20777bsup2.hkl

Structure factors: contains datablock(s) 7a. DOI: 10.1107/S2056989026003385/yz20777asup3.hkl

CCDC references: 2542751, 2542752

Additional supporting information:  crystallographic information; 3D view; checkCIF report

## Figures and Tables

**Figure 2:**
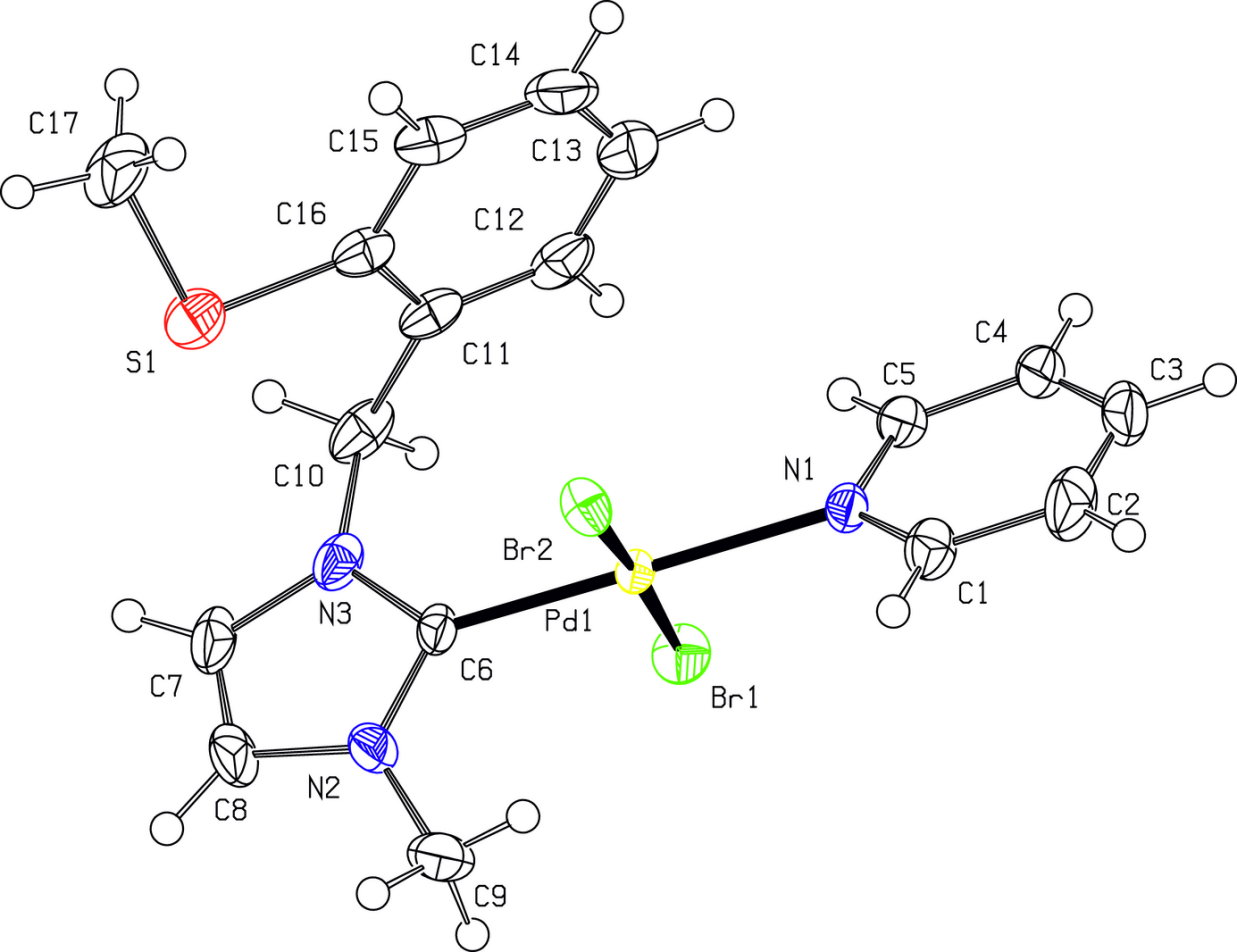
Mol­ecular structure of **7b**. Displacement ellipsoids are drawn at the 50% probability level; H atoms are given as spheres of arbitrary size.

**Table 1:** Selected geometric parameters (Å, °) for **7a**[Chem scheme1]

Pd1*A*—C6*A*	1.977 (6)	Pd1*B*—C6*B*	1.958 (6)
Pd1*A*—N1*A*	2.095 (5)	Pd1*B*—N1*B*	2.072 (5)
Pd1*A*—Cl2*A*	2.3077 (14)	Pd1*B*—Cl2*B*	2.3119 (15)
Pd1*A*—Cl1*A*	2.3137 (15)	Pd1*B*—Cl1*B*	2.3245 (15)
			
C6*A*—Pd1*A*—N1*A*	178.3 (2)	C6*B*—Pd1*B*—N1*B*	178.4 (2)
C6*A*—Pd1*A*—Cl2*A*	88.08 (15)	C6*B*—Pd1*B*—Cl2*B*	89.21 (18)
N1*A*—Pd1*A*—Cl2*A*	90.23 (14)	N1*B*—Pd1*B*—Cl2*B*	89.18 (14)
C6*A*—Pd1*A*—Cl1*A*	90.46 (15)	C6*B*—Pd1*B*—Cl1*B*	91.18 (18)
N1*A*—Pd1*A*—Cl1*A*	91.25 (14)	N1*B*—Pd1*B*—Cl1*B*	90.43 (14)
Cl2*A*—Pd1*A*—Cl1*A*	177.53 (6)	Cl2*B*—Pd1*B*—Cl1*B*	177.21 (5)

**Table 2:** Selected geometric parameters (Å, °) for **7b**[Chem scheme1]

Pd1—C6	1.961 (3)	Pd1—Br1	2.4234 (4)
Pd1—N1	2.090 (3)	Pd1—Br2	2.4393 (4)
			
C6—Pd1—N1	177.21 (13)	C6—Pd1—Br2	91.02 (10)
C6—Pd1—Br1	87.46 (10)	N1—Pd1—Br2	91.48 (8)
N1—Pd1—Br1	90.13 (8)	Br1—Pd1—Br2	175.732 (17)

**Table 3:** Hydrogen-bond geometry (Å, °) for **7a**[Chem scheme1] *Cg*1 is the centroid of the imidazolium ring in mol­ecule *A* and *Cg*4 is the centroid of the imidazolium ring in mol­ecule *B*.

*D*—H⋯*A*	*D*—H	H⋯*A*	*D*⋯*A*	*D*—H⋯*A*
C2*A*—H2*A*⋯Cl2*B*	0.95	2.66	3.570 (6)	161
C4*A*—H4*A*⋯Cl1*A*^i^	0.95	2.83	3.536 (8)	132
C8*A*—H8*A*⋯Cl2*A*^ii^	0.95	2.77	3.514 (6)	136
C2*B*—H2*B*⋯Cl1*B*^iii^	0.95	2.78	3.573 (8)	141
C4*B*—H4*B*⋯Cl2*A*^iv^	0.95	2.72	3.415 (6)	130
C8*B*—H8*B*⋯Cl1*A*^i^	0.95	2.92	3.710 (6)	142
C9*A*—H9*AB*⋯*Cg*1^ii^	0.98	2.86	3.745 (6)	150
C9*B*—H9*BB*⋯*Cg*4^v^	0.98	2.84	3.765 (6)	158

Symmetry codes: (i) 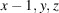; (ii) 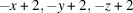; (iii) 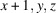; (iv) 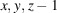; (v) 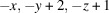.

**Table 4:** Hydrogen-bond geometry (Å, °) for **7b**[Chem scheme1] *Cg*3 is the centroid of the phenyl ring (C11–C16) of the (methyl­sulfan­yl)benzyl side arm.

*D*—H⋯*A*	*D*—H	H⋯*A*	*D*⋯*A*	*D*—H⋯*A*
C2—H2⋯Br1^i^	0.95	2.78	3.660 (4)	155
C4—H4⋯Br2^ii^	0.95	2.94	3.659 (4)	133
C8—H8⋯Br1^iii^	0.95	2.91	3.725 (4)	145
C10—H10*A*⋯*Cg*3^iv^	0.99	2.94	3.599 (4)	125

Symmetry codes: (i) 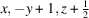; (ii) 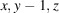; (iii) 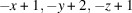; (iv) 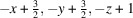.

**Table 5:** Experimental details

	**7a**	**7b**
Crystal data		
Chemical formula	[PdCl_2_(C_5_H_5_N)(C_12_H_14_N_2_S)]	[PdBr_2_(C_5_H_5_N)(C_12_H_14_N_2_S)]
*M*_r_	474.71	563.63
Crystal system, space group	Triclinic, *P* 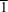	Monoclinic, *C*2/*c*
Temperature (K)	100	100
*a*, *b*, *c* (Å)	8.814 (2), 13.641 (3), 16.629 (4)	25.6614 (15), 8.9728 (5), 17.1373 (10)
α, β, γ (°)	94.423 (4), 91.909 (7), 108.238 (6)	90, 91.826 (2), 90
*V* (Å^3^)	1889.7 (8)	3943.9 (4)
*Z*	4	8
Radiation type	Mo *K*α	Mo *K*α
μ (mm^−1^)	1.38	5.10
Crystal size (mm)	0.10 × 0.10 × 0.07	0.12 × 0.12 × 0.06

Data collection		
Diffractometer	Bruker APEXII CCD	Bruker APEXII CCD
Absorption correction	Multi-scan (*TWINABS*; Bruker, 2020 ▸)	Multi-scan (*SADABS*; Krause *et al.*, 2015 ▸)
*T*_min_, *T*_max_	0.576, 0.746	0.522, 0.746
No. of measured, independent and observed [*I* > 2σ(*I*)] reflections	7994, 7994, 7000	37799, 7202, 5149
*R*_int_	–	0.062
(sin θ/λ)_max_ (Å^−1^)	0.740	0.764

Refinement		
*R*[*F*^2^ > 2σ(*F*^2^)], *wR*(*F*^2^), *S*	0.042, 0.121, 1.06	0.042, 0.083, 1.03
No. of reflections	7994	7202
No. of parameters	438	219
H-atom treatment	H-atom parameters constrained	H-atom parameters constrained
Δρ_max_, Δρ_min_ (e Å^−3^)	1.27, −1.23	1.04, −1.26

Computer programs: *APEX3* and *SAINT* (Bruker, 2020 ▸), *SHELXT* (Sheldrick, 2015*a* ▸), *SHELXL* (Sheldrick, 2015*b* ▸), *Mercury* (Macrae *et al.*, 2020 ▸), *PLATON* (Spek, 2020 ▸) and *publCIF* (Westrip, 2010 ▸).

## supplementary crystallographic information

### Dibromido{1-methyl-3-[2-(methylsulfanyl)benzyl]-2H-imidazol-2-ylidene-κC2}(pyridine-κN)palladium(II) (7b) Crystal data

**Table:**

[PdBr_2_(C_5_H_5_N)(C_12_H_14_N_2_S)]	*F*(000) = 2192
*M**_r_* = 563.63	*D*_x_ = 1.898 Mg m^−^^3^
Monoclinic, *C*2/*c*	Mo *K*α radiation, λ = 0.71073 Å
*a* = 25.6614 (15) Å	Cell parameters from 8216 reflections
*b* = 8.9728 (5) Å	θ = 2.4–30.2°
*c* = 17.1373 (10) Å	µ = 5.10 mm^−^^1^
β = 91.826 (2)°	*T* = 100 K
*V* = 3943.9 (4) Å^3^	Plate, orange
*Z* = 8	0.12 × 0.12 × 0.06 mm

### Dibromido{1-methyl-3-[2-(methylsulfanyl)benzyl]-2H-imidazol-2-ylidene-κC2}(pyridine-κN)palladium(II) (7b) Data collection

**Table:**

Bruker APEXII CCD diffractometer	5149 reflections with *I* > 2σ(*I*)
ω– and φ–scans	*R*_int_ = 0.062
Absorption correction: multi-scan (SADABS; Krause *et al.*, 2015)	θ_max_ = 32.9°, θ_min_ = 2.9°
*T*_min_ = 0.522, *T*_max_ = 0.746	*h* = −38→38
37799 measured reflections	*k* = −13→13
7202 independent reflections	*l* = −25→24

### Dibromido{1-methyl-3-[2-(methylsulfanyl)benzyl]-2H-imidazol-2-ylidene-κC2}(pyridine-κN)palladium(II) (7b) Refinement

**Table:**

Refinement on *F*^2^	Primary atom site location: dual
Least-squares matrix: full	Secondary atom site location: difference Fourier map
*R*[*F*^2^ > 2σ(*F*^2^)] = 0.042	Hydrogen site location: inferred from neighbouring sites
*wR*(*F*^2^) = 0.083	H-atom parameters constrained
*S* = 1.03	*w* = 1/[σ^2^(*F*_o_^2^) + (0.020*P*)^2^ + 17.3873*P*] where *P* = (*F*_o_^2^ + 2*F*_c_^2^)/3
7202 reflections	(Δ/σ)_max_ = 0.001
219 parameters	Δρ_max_ = 1.04 e Å^−^^3^
0 restraints	Δρ_min_ = −1.26 e Å^−^^3^

### Dibromido{1-methyl-3-[2-(methylsulfanyl)benzyl]-2H-imidazol-2-ylidene-κC2}(pyridine-κN)palladium(II) (7b) Special details

**Table:**

Geometry. All esds (except the esd in the dihedral angle between two l.s. planes) are estimated using the full covariance matrix. The cell esds are taken into account individually in the estimation of esds in distances, angles and torsion angles; correlations between esds in cell parameters are only used when they are defined by crystal symmetry. An approximate (isotropic) treatment of cell esds is used for estimating esds involving l.s. planes.

### Dibromido{1-methyl-3-[2-(methylsulfanyl)benzyl]-2H-imidazol-2-ylidene-κC2}(pyridine-κN)palladium(II) (7b) Fractional atomic coordinates and isotropic or equivalent isotropic displacement parameters (Å^2^)

**Table:**

	*x*	*y*	*z*	*U*_iso_*/*U*_eq_	
Pd1	0.58794 (2)	0.76275 (3)	0.65280 (2)	0.01636 (6)	
Br1	0.54663 (2)	0.62727 (4)	0.54527 (2)	0.02871 (9)	
Br2	0.62357 (2)	0.90994 (4)	0.76163 (2)	0.02476 (8)	
S1	0.72809 (4)	1.08861 (12)	0.58287 (6)	0.0363 (2)	
N1	0.58644 (11)	0.5710 (3)	0.72190 (16)	0.0214 (6)	
N2	0.55725 (12)	1.0543 (3)	0.58191 (18)	0.0285 (7)	
N3	0.62193 (11)	0.9573 (4)	0.52308 (16)	0.0282 (7)	
C1	0.56575 (15)	0.5676 (4)	0.7922 (2)	0.0287 (8)	
H1	0.550563	0.655770	0.812060	0.034*	
C2	0.56575 (17)	0.4385 (5)	0.8374 (2)	0.0414 (10)	
H2	0.550574	0.438631	0.887204	0.050*	
C3	0.58790 (16)	0.3111 (5)	0.8091 (3)	0.0405 (10)	
H3	0.588848	0.222689	0.839540	0.049*	
C4	0.60864 (14)	0.3128 (4)	0.7364 (2)	0.0331 (9)	
H4	0.623881	0.225575	0.715464	0.040*	
C5	0.60691 (14)	0.4441 (4)	0.6940 (2)	0.0278 (7)	
H5	0.620722	0.444916	0.643222	0.033*	
C6	0.58962 (13)	0.9364 (4)	0.58350 (18)	0.0216 (7)	
C7	0.60892 (16)	1.0889 (5)	0.4849 (2)	0.0402 (11)	
H7	0.625758	1.128787	0.441012	0.048*	
C8	0.56905 (17)	1.1493 (5)	0.5202 (2)	0.0406 (10)	
H8	0.551774	1.239589	0.506382	0.049*	
C9	0.51423 (15)	1.0759 (5)	0.6339 (2)	0.0370 (9)	
H9A	0.511459	1.181770	0.647118	0.055*	
H9B	0.520505	1.017760	0.681651	0.055*	
H9C	0.481709	1.042669	0.607835	0.055*	
C10	0.66222 (15)	0.8526 (5)	0.4986 (2)	0.0338 (9)	
H10A	0.683687	0.901849	0.459166	0.041*	
H10B	0.645015	0.765575	0.473334	0.041*	
C11	0.69754 (14)	0.7981 (5)	0.5643 (2)	0.0298 (8)	
C12	0.69970 (15)	0.6466 (5)	0.5813 (2)	0.0344 (9)	
H12	0.678172	0.579415	0.551984	0.041*	
C13	0.73286 (16)	0.5914 (5)	0.6404 (3)	0.0408 (10)	
H13	0.733816	0.487744	0.651622	0.049*	
C14	0.76420 (15)	0.6889 (5)	0.6825 (3)	0.0407 (10)	
H14	0.786552	0.651834	0.723237	0.049*	
C15	0.76371 (15)	0.8401 (5)	0.6663 (2)	0.0359 (9)	
H15	0.785997	0.905607	0.695411	0.043*	
C16	0.73036 (14)	0.8967 (4)	0.6071 (2)	0.0285 (8)	
C17	0.79145 (17)	1.1551 (6)	0.6165 (3)	0.0488 (12)	
H17A	0.818664	1.092477	0.594679	0.073*	
H17B	0.793911	1.150428	0.673668	0.073*	
H17C	0.796088	1.258364	0.599502	0.073*	

### Dibromido{1-methyl-3-[2-(methylsulfanyl)benzyl]-2H-imidazol-2-ylidene-κC2}(pyridine-κN)palladium(II) (7b) Atomic displacement parameters (Å^2^)

**Table:**

	*U*^11^	*U*^22^	*U*^33^	*U*^12^	*U*^13^	*U*^23^
Pd1	0.01711 (11)	0.01611 (12)	0.01588 (10)	−0.00196 (9)	0.00101 (8)	0.00103 (9)
Br1	0.0307 (2)	0.0304 (2)	0.02467 (17)	−0.00643 (15)	−0.00623 (14)	−0.00430 (14)
Br2	0.03307 (19)	0.01896 (16)	0.02189 (16)	−0.00091 (14)	−0.00493 (13)	−0.00191 (12)
S1	0.0297 (5)	0.0352 (5)	0.0436 (6)	−0.0077 (4)	−0.0057 (4)	0.0016 (4)
N1	0.0206 (14)	0.0212 (14)	0.0223 (13)	−0.0046 (11)	−0.0020 (10)	0.0044 (11)
N2	0.0240 (15)	0.0243 (15)	0.0366 (17)	−0.0011 (12)	−0.0083 (12)	0.0088 (13)
N3	0.0238 (15)	0.0394 (18)	0.0211 (14)	−0.0099 (13)	−0.0027 (11)	0.0079 (13)
C1	0.033 (2)	0.032 (2)	0.0218 (16)	−0.0046 (16)	0.0010 (14)	0.0034 (14)
C2	0.044 (2)	0.051 (3)	0.029 (2)	−0.013 (2)	0.0002 (17)	0.0168 (19)
C3	0.034 (2)	0.033 (2)	0.054 (3)	−0.0139 (18)	−0.0140 (19)	0.0206 (19)
C4	0.0237 (19)	0.0238 (19)	0.051 (2)	−0.0055 (15)	−0.0054 (16)	0.0094 (17)
C5	0.0233 (18)	0.0225 (18)	0.0375 (19)	−0.0024 (14)	−0.0008 (14)	0.0027 (15)
C6	0.0207 (16)	0.0229 (17)	0.0210 (15)	−0.0076 (13)	−0.0045 (12)	0.0065 (12)
C7	0.033 (2)	0.049 (3)	0.038 (2)	−0.0185 (19)	−0.0125 (17)	0.0258 (19)
C8	0.036 (2)	0.038 (2)	0.046 (2)	−0.0092 (18)	−0.0200 (18)	0.0246 (19)
C9	0.026 (2)	0.033 (2)	0.052 (2)	0.0071 (16)	−0.0051 (17)	0.0010 (18)
C10	0.0280 (19)	0.054 (3)	0.0198 (16)	−0.0123 (18)	0.0062 (14)	−0.0043 (16)
C11	0.0189 (17)	0.042 (2)	0.0290 (18)	−0.0017 (15)	0.0095 (14)	−0.0048 (16)
C12	0.0236 (19)	0.038 (2)	0.043 (2)	−0.0037 (16)	0.0143 (16)	−0.0080 (18)
C13	0.032 (2)	0.038 (2)	0.053 (3)	0.0060 (18)	0.018 (2)	0.006 (2)
C14	0.0213 (19)	0.054 (3)	0.047 (2)	0.0108 (19)	0.0041 (17)	0.005 (2)
C15	0.0209 (19)	0.048 (3)	0.038 (2)	0.0022 (17)	−0.0016 (16)	−0.0033 (18)
C16	0.0200 (17)	0.039 (2)	0.0272 (17)	0.0002 (15)	0.0053 (14)	−0.0032 (15)
C17	0.037 (2)	0.052 (3)	0.057 (3)	−0.025 (2)	−0.010 (2)	0.004 (2)

### Dibromido{1-methyl-3-[2-(methylsulfanyl)benzyl]-2H-imidazol-2-ylidene-κC2}(pyridine-κN)palladium(II) (7b) Geometric parameters (Å, º)

**Table:**

Pd1—C6	1.961 (3)	C7—C8	1.321 (6)
Pd1—N1	2.090 (3)	C7—H7	0.9500
Pd1—Br1	2.4234 (4)	C8—H8	0.9500
Pd1—Br2	2.4393 (4)	C9—H9A	0.9800
S1—C16	1.772 (4)	C9—H9B	0.9800
S1—C17	1.809 (4)	C9—H9C	0.9800
N1—C1	1.332 (4)	C10—C11	1.504 (5)
N1—C5	1.347 (5)	C10—H10A	0.9900
N2—C6	1.345 (5)	C10—H10B	0.9900
N2—C8	1.399 (5)	C11—C12	1.391 (6)
N2—C9	1.453 (5)	C11—C16	1.411 (5)
N3—C6	1.360 (4)	C12—C13	1.393 (6)
N3—C7	1.385 (5)	C12—H12	0.9500
N3—C10	1.468 (5)	C13—C14	1.376 (6)
C1—C2	1.394 (5)	C13—H13	0.9500
C1—H1	0.9500	C14—C15	1.385 (6)
C2—C3	1.372 (6)	C14—H14	0.9500
C2—H2	0.9500	C15—C16	1.402 (5)
C3—C4	1.370 (6)	C15—H15	0.9500
C3—H3	0.9500	C17—H17A	0.9800
C4—C5	1.384 (5)	C17—H17B	0.9800
C4—H4	0.9500	C17—H17C	0.9800
C5—H5	0.9500		
			
C6—Pd1—N1	177.21 (13)	C7—C8—H8	126.7
C6—Pd1—Br1	87.46 (10)	N2—C8—H8	126.7
N1—Pd1—Br1	90.13 (8)	N2—C9—H9A	109.5
C6—Pd1—Br2	91.02 (10)	N2—C9—H9B	109.5
N1—Pd1—Br2	91.48 (8)	H9A—C9—H9B	109.5
Br1—Pd1—Br2	175.732 (17)	N2—C9—H9C	109.5
C16—S1—C17	102.9 (2)	H9A—C9—H9C	109.5
C1—N1—C5	118.2 (3)	H9B—C9—H9C	109.5
C1—N1—Pd1	123.1 (3)	N3—C10—C11	114.1 (3)
C5—N1—Pd1	118.7 (2)	N3—C10—H10A	108.7
C6—N2—C8	110.2 (3)	C11—C10—H10A	108.7
C6—N2—C9	125.0 (3)	N3—C10—H10B	108.7
C8—N2—C9	124.7 (3)	C11—C10—H10B	108.7
C6—N3—C7	109.4 (3)	H10A—C10—H10B	107.6
C6—N3—C10	125.5 (3)	C12—C11—C16	119.0 (4)
C7—N3—C10	124.9 (3)	C12—C11—C10	119.5 (4)
N1—C1—C2	121.9 (4)	C16—C11—C10	121.4 (4)
N1—C1—H1	119.1	C11—C12—C13	121.2 (4)
C2—C1—H1	119.1	C11—C12—H12	119.4
C3—C2—C1	119.3 (4)	C13—C12—H12	119.4
C3—C2—H2	120.4	C14—C13—C12	119.2 (4)
C1—C2—H2	120.4	C14—C13—H13	120.4
C4—C3—C2	119.3 (4)	C12—C13—H13	120.4
C4—C3—H3	120.3	C13—C14—C15	121.1 (4)
C2—C3—H3	120.3	C13—C14—H14	119.4
C3—C4—C5	118.6 (4)	C15—C14—H14	119.4
C3—C4—H4	120.7	C14—C15—C16	120.0 (4)
C5—C4—H4	120.7	C14—C15—H15	120.0
N1—C5—C4	122.6 (4)	C16—C15—H15	120.0
N1—C5—H5	118.7	C15—C16—C11	119.4 (4)
C4—C5—H5	118.7	C15—C16—S1	122.4 (3)
N2—C6—N3	105.5 (3)	C11—C16—S1	118.2 (3)
N2—C6—Pd1	127.7 (2)	S1—C17—H17A	109.5
N3—C6—Pd1	126.6 (3)	S1—C17—H17B	109.5
C8—C7—N3	108.2 (3)	H17A—C17—H17B	109.5
C8—C7—H7	125.9	S1—C17—H17C	109.5
N3—C7—H7	125.9	H17A—C17—H17C	109.5
C7—C8—N2	106.6 (4)	H17B—C17—H17C	109.5
			
C5—N1—C1—C2	1.3 (5)	C6—N2—C8—C7	−0.5 (4)
Pd1—N1—C1—C2	−179.3 (3)	C9—N2—C8—C7	−177.1 (4)
N1—C1—C2—C3	0.4 (6)	C6—N3—C10—C11	−49.4 (5)
C1—C2—C3—C4	−1.3 (6)	C7—N3—C10—C11	134.8 (4)
C2—C3—C4—C5	0.6 (6)	N3—C10—C11—C12	119.3 (4)
C1—N1—C5—C4	−2.0 (5)	N3—C10—C11—C16	−63.5 (4)
Pd1—N1—C5—C4	178.6 (3)	C16—C11—C12—C13	1.2 (5)
C3—C4—C5—N1	1.1 (6)	C10—C11—C12—C13	178.5 (3)
C8—N2—C6—N3	0.2 (4)	C11—C12—C13—C14	−0.4 (6)
C9—N2—C6—N3	176.9 (3)	C12—C13—C14—C15	−0.7 (6)
C8—N2—C6—Pd1	−175.7 (3)	C13—C14—C15—C16	1.0 (6)
C9—N2—C6—Pd1	1.0 (5)	C14—C15—C16—C11	−0.1 (6)
C7—N3—C6—N2	0.1 (4)	C14—C15—C16—S1	−179.5 (3)
C10—N3—C6—N2	−176.3 (3)	C12—C11—C16—C15	−0.9 (5)
C7—N3—C6—Pd1	176.0 (3)	C10—C11—C16—C15	−178.2 (3)
C10—N3—C6—Pd1	−0.4 (5)	C12—C11—C16—S1	178.5 (3)
C6—N3—C7—C8	−0.4 (4)	C10—C11—C16—S1	1.3 (5)
C10—N3—C7—C8	176.1 (3)	C17—S1—C16—C15	23.7 (4)
N3—C7—C8—N2	0.5 (5)	C17—S1—C16—C11	−155.7 (3)

### Dibromido{1-methyl-3-[2-(methylsulfanyl)benzyl]-2H-imidazol-2-ylidene-κC2}(pyridine-κN)palladium(II) (7b) Hydrogen-bond geometry (Å, º)

*Cg*3 is the centroid of the phenyl ring (C11–C16) of the (methylsulfanyl)benzyl side arm.

**Table:**

*D*—H···*A*	*D*—H	H···*A*	*D*···*A*	*D*—H···*A*
C2—H2···Br1^i^	0.95	2.78	3.660 (4)	155
C4—H4···Br2^ii^	0.95	2.94	3.659 (4)	133
C8—H8···Br1^iii^	0.95	2.91	3.725 (4)	145
C10—H10*A*···*Cg*3^iv^	0.99	2.94	3.599 (4)	125

Symmetry codes: (i) *x*, −*y*+1, *z*+1/2; (ii) *x*, *y*−1, *z*; (iii) −*x*+1, −*y*+2, −*z*+1; (iv) −*x*+3/2, −*y*+3/2, −*z*+1.

### Dichlorido{1-methyl-3-[2-(methylsulfanyl)benzyl]-2H-imidazol-2-ylidene-κC2}(pyridine-κN)palladium(II) (7a) Crystal data

**Table:**

[PdCl_2_(C_5_H_5_N)(C_12_H_14_N_2_S)]	*Z* = 4
*M**_r_* = 474.71	*F*(000) = 952
Triclinic, *P*1	*D*_x_ = 1.669 Mg m^−^^3^
*a* = 8.814 (2) Å	Mo *K*α radiation, λ = 0.71073 Å
*b* = 13.641 (3) Å	Cell parameters from 3261 reflections
*c* = 16.629 (4) Å	θ = 2.4–31.0°
α = 94.423 (4)°	µ = 1.38 mm^−^^1^
β = 91.909 (7)°	*T* = 100 K
γ = 108.238 (6)°	Fragment, green tinged yellow
*V* = 1889.7 (8) Å^3^	0.10 × 0.10 × 0.07 mm

### Dichlorido{1-methyl-3-[2-(methylsulfanyl)benzyl]-2H-imidazol-2-ylidene-κC2}(pyridine-κN)palladium(II) (7a) Data collection

**Table:**

Bruker APEXII CCD diffractometer	7000 reflections with *I* > 2σ(*I*)
ω– and φ–scans	θ_max_ = 31.7°, θ_min_ = 1.9°
Absorption correction: multi-scan (*TWINABS*; Bruker, 2020)	*h* = −11→11
*T*_min_ = 0.576, *T*_max_ = 0.746	*k* = −20→19
7994 measured reflections	*l* = 0→24
7994 independent reflections	

### Dichlorido{1-methyl-3-[2-(methylsulfanyl)benzyl]-2H-imidazol-2-ylidene-κC2}(pyridine-κN)palladium(II) (7a) Refinement

**Table:**

Refinement on *F*^2^	Primary atom site location: dual
Least-squares matrix: full	Secondary atom site location: difference Fourier map
*R*[*F*^2^ > 2σ(*F*^2^)] = 0.042	Hydrogen site location: inferred from neighbouring sites
*wR*(*F*^2^) = 0.121	H-atom parameters constrained
*S* = 1.06	*w* = 1/[σ^2^(*F*_o_^2^) + (0.0753*P*)^2^ + 1.1948*P*] where *P* = (*F*_o_^2^ + 2*F*_c_^2^)/3
7994 reflections	(Δ/σ)_max_ = 0.001
438 parameters	Δρ_max_ = 1.27 e Å^−^^3^
0 restraints	Δρ_min_ = −1.23 e Å^−^^3^

### Dichlorido{1-methyl-3-[2-(methylsulfanyl)benzyl]-2H-imidazol-2-ylidene-κC2}(pyridine-κN)palladium(II) (7a) Special details

**Table:**

Geometry. All esds (except the esd in the dihedral angle between two l.s. planes) are estimated using the full covariance matrix. The cell esds are taken into account individually in the estimation of esds in distances, angles and torsion angles; correlations between esds in cell parameters are only used when they are defined by crystal symmetry. An approximate (isotropic) treatment of cell esds is used for estimating esds involving l.s. planes.
Refinement. Refined as a 2-component twin.

### Dichlorido{1-methyl-3-[2-(methylsulfanyl)benzyl]-2H-imidazol-2-ylidene-κC2}(pyridine-κN)palladium(II) (7a) Fractional atomic coordinates and isotropic or equivalent isotropic displacement parameters (Å^2^)

**Table:**

	*x*	*y*	*z*	*U*_iso_*/*U*_eq_	
Pd1A	0.67792 (6)	0.82214 (3)	0.85685 (2)	0.01392 (13)	
Cl1A	0.78256 (19)	0.75871 (12)	0.74618 (8)	0.0205 (3)	
Cl2A	0.58378 (19)	0.89119 (11)	0.96818 (8)	0.0165 (3)	
S1A	0.8781 (2)	0.54356 (12)	0.91419 (9)	0.0246 (3)	
N1A	0.4751 (7)	0.8203 (4)	0.7865 (3)	0.0180 (11)	
N2A	1.0148 (7)	0.8895 (4)	0.9256 (3)	0.0162 (10)	
N3A	0.8673 (7)	0.7627 (3)	0.9858 (3)	0.0164 (10)	
C1A	0.4856 (9)	0.8566 (5)	0.7126 (3)	0.0241 (14)	
H1A	0.587788	0.881845	0.691288	0.029*	
C2A	0.3516 (10)	0.8581 (5)	0.6673 (3)	0.0306 (16)	
H2A	0.361680	0.884235	0.615679	0.037*	
C3A	0.2026 (10)	0.8208 (5)	0.6985 (4)	0.0314 (16)	
H3A	0.108847	0.821136	0.668862	0.038*	
C4A	0.1930 (9)	0.7836 (4)	0.7729 (4)	0.0248 (14)	
H4A	0.091622	0.756331	0.794701	0.030*	
C5A	0.3295 (8)	0.7854 (4)	0.8163 (3)	0.0204 (13)	
H5A	0.321006	0.761395	0.868616	0.025*	
C6A	0.8660 (7)	0.8247 (4)	0.9262 (3)	0.0114 (11)	
C7A	1.0220 (9)	0.7903 (4)	1.0229 (3)	0.0224 (14)	
H7A	1.054876	0.759219	1.066599	0.027*	
C8A	1.1155 (9)	0.8694 (4)	0.9848 (3)	0.0219 (13)	
H8A	1.226634	0.904276	0.995844	0.026*	
C9A	1.0666 (8)	0.9741 (4)	0.8728 (3)	0.0213 (13)	
H9AA	1.160818	0.969161	0.845220	0.032*	
H9AB	1.093753	1.040908	0.905462	0.032*	
H9AC	0.979791	0.968698	0.832645	0.032*	
C10A	0.7289 (8)	0.6828 (4)	1.0128 (3)	0.0192 (12)	
H10A	0.663508	0.717355	1.044265	0.023*	
H10B	0.768101	0.640988	1.049548	0.023*	
C11A	0.6225 (8)	0.6103 (5)	0.9449 (3)	0.0176 (13)	
C12A	0.6785 (8)	0.5421 (5)	0.8959 (4)	0.0186 (13)	
C13A	0.5727 (9)	0.4743 (5)	0.8346 (4)	0.0229 (15)	
H13A	0.608203	0.427855	0.800353	0.027*	
C14A	0.4141 (10)	0.4766 (6)	0.8250 (4)	0.0277 (17)	
H14A	0.342694	0.431527	0.784081	0.033*	
C15A	0.3631 (9)	0.5424 (5)	0.8738 (4)	0.0275 (16)	
H15A	0.255751	0.542685	0.867186	0.033*	
C16A	0.4662 (9)	0.6096 (5)	0.9334 (4)	0.0222 (15)	
H16A	0.428687	0.655724	0.966844	0.027*	
C17A	0.8722 (10)	0.4160 (4)	0.8718 (4)	0.0281 (15)	
H17A	0.860685	0.412331	0.812702	0.042*	
H17B	0.781133	0.363600	0.891648	0.042*	
H17C	0.971714	0.403182	0.888026	0.042*	
Pd1B	0.12773 (6)	0.82757 (3)	0.35135 (2)	0.01539 (14)	
Cl1B	−0.0573 (2)	0.76562 (12)	0.24165 (8)	0.0214 (3)	
Cl2B	0.3138 (2)	0.89727 (11)	0.45865 (8)	0.0201 (3)	
S1B	−0.3215 (2)	0.54588 (12)	0.41477 (10)	0.0266 (4)	
N1B	0.3134 (7)	0.8341 (3)	0.2766 (3)	0.0159 (10)	
N2B	−0.1345 (7)	0.8853 (4)	0.4293 (3)	0.0201 (11)	
N3B	−0.0883 (7)	0.7601 (4)	0.4846 (3)	0.0181 (11)	
C1B	0.4255 (9)	0.7916 (5)	0.2972 (4)	0.0251 (15)	
H1B	0.417066	0.758871	0.346013	0.030*	
C2B	0.5535 (9)	0.7933 (5)	0.2503 (4)	0.0259 (14)	
H2B	0.629760	0.761108	0.265833	0.031*	
C3B	0.5671 (9)	0.8432 (5)	0.1800 (4)	0.0284 (16)	
H3B	0.653696	0.846402	0.146737	0.034*	
C4B	0.4509 (9)	0.8891 (5)	0.1589 (3)	0.0265 (15)	
H4B	0.456898	0.923692	0.111060	0.032*	
C5B	0.3300 (9)	0.8824 (4)	0.2087 (3)	0.0208 (13)	
H5B	0.252062	0.914028	0.194513	0.025*	
C6B	−0.0433 (8)	0.8233 (4)	0.4245 (3)	0.0160 (12)	
C7B	−0.2383 (9)	0.8621 (5)	0.4907 (4)	0.0264 (15)	
H7B	−0.316003	0.894259	0.505141	0.032*	
C8B	−0.2065 (9)	0.7841 (5)	0.5257 (4)	0.0263 (15)	
H8B	−0.256784	0.751710	0.570737	0.032*	
C9B	−0.1243 (9)	0.9699 (4)	0.3779 (3)	0.0241 (13)	
H9BA	−0.089638	0.952542	0.324735	0.036*	
H9BB	−0.046811	1.034122	0.403139	0.036*	
H9BC	−0.229597	0.979354	0.371434	0.036*	
C10B	−0.0141 (9)	0.6829 (4)	0.5076 (3)	0.0248 (14)	
H10C	−0.079159	0.641707	0.548038	0.030*	
H10D	0.093314	0.719863	0.533848	0.030*	
C11B	0.0034 (9)	0.6094 (5)	0.4381 (3)	0.0194 (13)	
C12B	0.1571 (9)	0.6066 (5)	0.4233 (4)	0.0234 (14)	
H12B	0.246655	0.652569	0.454762	0.028*	
C13B	0.1804 (9)	0.5374 (5)	0.3629 (4)	0.0266 (15)	
H13B	0.285137	0.535621	0.353106	0.032*	
C14B	0.0463 (10)	0.4697 (5)	0.3162 (4)	0.0270 (16)	
H14B	0.060901	0.422725	0.274299	0.032*	
C15B	−0.1051 (10)	0.4716 (5)	0.3314 (4)	0.0260 (16)	
H15B	−0.194780	0.425565	0.300050	0.031*	
C16B	−0.1281 (9)	0.5413 (5)	0.3929 (3)	0.0183 (13)	
C17B	−0.4514 (10)	0.4176 (5)	0.3779 (4)	0.0357 (18)	
H17D	−0.443145	0.406304	0.319505	0.054*	
H17E	−0.562280	0.411565	0.389065	0.054*	
H17F	−0.419076	0.365452	0.405171	0.054*	

### Dichlorido{1-methyl-3-[2-(methylsulfanyl)benzyl]-2H-imidazol-2-ylidene-κC2}(pyridine-κN)palladium(II) (7a) Atomic displacement parameters (Å^2^)

**Table:**

	*U*^11^	*U*^22^	*U*^33^	*U*^12^	*U*^13^	*U*^23^
Pd1A	0.0145 (3)	0.01269 (19)	0.01437 (17)	0.00451 (19)	−0.00069 (16)	−0.00038 (14)
Cl1A	0.0148 (8)	0.0296 (7)	0.0176 (5)	0.0095 (6)	0.0000 (5)	−0.0052 (5)
Cl2A	0.0140 (8)	0.0175 (6)	0.0184 (5)	0.0064 (6)	0.0021 (5)	−0.0027 (4)
S1A	0.0214 (10)	0.0195 (7)	0.0333 (7)	0.0087 (8)	−0.0022 (7)	−0.0033 (6)
N1A	0.021 (3)	0.015 (2)	0.018 (2)	0.008 (2)	−0.004 (2)	−0.0037 (17)
N2A	0.015 (3)	0.016 (2)	0.020 (2)	0.008 (2)	−0.0021 (18)	0.0005 (17)
N3A	0.017 (3)	0.009 (2)	0.022 (2)	0.003 (2)	−0.0008 (19)	−0.0014 (17)
C1A	0.027 (4)	0.033 (3)	0.018 (2)	0.018 (3)	0.003 (2)	−0.001 (2)
C2A	0.040 (5)	0.039 (4)	0.017 (2)	0.021 (4)	−0.005 (3)	−0.002 (2)
C3A	0.034 (5)	0.029 (3)	0.034 (3)	0.018 (3)	−0.014 (3)	−0.012 (2)
C4A	0.019 (4)	0.012 (2)	0.042 (3)	0.007 (3)	−0.008 (3)	−0.010 (2)
C5A	0.021 (4)	0.016 (3)	0.024 (2)	0.007 (3)	−0.010 (2)	−0.005 (2)
C6A	0.011 (3)	0.011 (2)	0.012 (2)	0.005 (2)	−0.0016 (19)	−0.0060 (17)
C7A	0.027 (4)	0.015 (3)	0.024 (3)	0.008 (3)	−0.011 (3)	−0.001 (2)
C8A	0.015 (3)	0.016 (3)	0.031 (3)	0.002 (3)	−0.006 (2)	−0.006 (2)
C9A	0.022 (4)	0.019 (3)	0.020 (2)	0.002 (3)	0.002 (2)	0.002 (2)
C10A	0.022 (4)	0.015 (2)	0.021 (2)	0.005 (3)	0.004 (2)	0.0014 (19)
C11A	0.017 (3)	0.013 (3)	0.020 (2)	0.001 (3)	0.000 (2)	0.003 (2)
C12A	0.014 (3)	0.011 (3)	0.031 (3)	0.004 (3)	0.006 (2)	0.003 (2)
C13A	0.024 (4)	0.015 (3)	0.030 (3)	0.008 (3)	0.000 (3)	−0.001 (2)
C14A	0.021 (4)	0.022 (3)	0.033 (3)	−0.002 (3)	−0.003 (3)	0.004 (3)
C15A	0.012 (4)	0.021 (3)	0.048 (4)	0.001 (3)	0.001 (3)	0.015 (3)
C16A	0.025 (4)	0.012 (3)	0.030 (3)	0.005 (3)	0.006 (3)	0.009 (2)
C17A	0.029 (4)	0.013 (2)	0.042 (3)	0.006 (3)	0.003 (3)	0.000 (2)
Pd1B	0.0172 (4)	0.01334 (19)	0.01391 (17)	0.00278 (18)	0.00255 (16)	−0.00116 (14)
Cl1B	0.0169 (8)	0.0277 (7)	0.0182 (5)	0.0064 (7)	0.0008 (5)	−0.0039 (5)
Cl2B	0.0189 (8)	0.0201 (6)	0.0183 (5)	0.0037 (6)	−0.0023 (5)	−0.0042 (5)
S1B	0.0231 (10)	0.0176 (7)	0.0352 (8)	0.0027 (7)	−0.0003 (7)	−0.0048 (6)
N1B	0.019 (3)	0.011 (2)	0.0170 (18)	0.004 (2)	0.0013 (19)	−0.0045 (15)
N2B	0.016 (3)	0.015 (2)	0.027 (2)	0.002 (2)	0.009 (2)	−0.0048 (18)
N3B	0.017 (3)	0.016 (2)	0.0165 (19)	0.000 (2)	0.0048 (19)	−0.0032 (17)
C1B	0.032 (4)	0.020 (3)	0.026 (3)	0.012 (3)	0.009 (3)	0.000 (2)
C2B	0.013 (4)	0.020 (3)	0.043 (3)	0.006 (3)	0.003 (3)	−0.005 (2)
C3B	0.028 (4)	0.022 (3)	0.031 (3)	0.002 (3)	0.018 (3)	−0.009 (2)
C4B	0.027 (4)	0.026 (3)	0.021 (2)	0.001 (3)	0.004 (3)	−0.001 (2)
C5B	0.021 (4)	0.016 (2)	0.022 (2)	0.002 (3)	0.000 (2)	−0.0014 (19)
C6B	0.012 (3)	0.012 (2)	0.019 (2)	−0.004 (2)	0.003 (2)	0.0001 (19)
C7B	0.019 (4)	0.023 (3)	0.034 (3)	0.004 (3)	0.012 (3)	−0.008 (2)
C8B	0.026 (4)	0.021 (3)	0.027 (3)	0.000 (3)	0.013 (3)	−0.005 (2)
C9B	0.024 (4)	0.023 (3)	0.031 (3)	0.016 (3)	0.002 (3)	−0.001 (2)
C10B	0.034 (4)	0.017 (3)	0.017 (2)	0.000 (3)	0.001 (3)	0.0016 (19)
C11B	0.023 (4)	0.015 (3)	0.021 (2)	0.007 (3)	0.002 (3)	0.004 (2)
C12B	0.023 (4)	0.017 (3)	0.029 (3)	0.004 (3)	0.001 (3)	0.008 (2)
C13B	0.022 (4)	0.016 (3)	0.039 (3)	0.001 (3)	0.003 (3)	0.010 (3)
C14B	0.036 (5)	0.016 (3)	0.027 (3)	0.007 (3)	0.001 (3)	−0.001 (2)
C15B	0.033 (5)	0.016 (3)	0.028 (3)	0.008 (3)	−0.001 (3)	−0.002 (2)
C16B	0.019 (4)	0.017 (3)	0.019 (2)	0.006 (3)	−0.007 (2)	0.001 (2)
C17B	0.032 (4)	0.020 (3)	0.044 (4)	−0.006 (3)	−0.002 (3)	−0.001 (3)

### Dichlorido{1-methyl-3-[2-(methylsulfanyl)benzyl]-2H-imidazol-2-ylidene-κC2}(pyridine-κN)palladium(II) (7a) Geometric parameters (Å, º)

**Table:**

Pd1A—C6A	1.977 (6)	Pd1B—C6B	1.958 (6)
Pd1A—N1A	2.095 (5)	Pd1B—N1B	2.072 (5)
Pd1A—Cl2A	2.3077 (14)	Pd1B—Cl2B	2.3119 (15)
Pd1A—Cl1A	2.3137 (15)	Pd1B—Cl1B	2.3245 (15)
S1A—C12A	1.769 (7)	S1B—C16B	1.774 (7)
S1A—C17A	1.810 (6)	S1B—C17B	1.812 (6)
N1A—C5A	1.347 (9)	N1B—C1B	1.340 (9)
N1A—C1A	1.355 (7)	N1B—C5B	1.341 (7)
N2A—C6A	1.334 (8)	N2B—C6B	1.337 (8)
N2A—C8A	1.402 (8)	N2B—C7B	1.383 (7)
N2A—C9A	1.471 (6)	N2B—C9B	1.470 (7)
N3A—C6A	1.354 (6)	N3B—C6B	1.358 (6)
N3A—C7A	1.402 (9)	N3B—C8B	1.374 (8)
N3A—C10A	1.469 (7)	N3B—C10B	1.470 (8)
C1A—C2A	1.387 (10)	C1B—C2B	1.388 (9)
C1A—H1A	0.9500	C1B—H1B	0.9500
C2A—C3A	1.386 (11)	C2B—C3B	1.387 (9)
C2A—H2A	0.9500	C2B—H2B	0.9500
C3A—C4A	1.369 (9)	C3B—C4B	1.405 (11)
C3A—H3A	0.9500	C3B—H3B	0.9500
C4A—C5A	1.374 (9)	C4B—C5B	1.357 (9)
C4A—H4A	0.9500	C4B—H4B	0.9500
C5A—H5A	0.9500	C5B—H5B	0.9500
C7A—C8A	1.353 (8)	C7B—C8B	1.349 (9)
C7A—H7A	0.9500	C7B—H7B	0.9500
C8A—H8A	0.9500	C8B—H8B	0.9500
C9A—H9AA	0.9800	C9B—H9BA	0.9800
C9A—H9AB	0.9800	C9B—H9BB	0.9800
C9A—H9AC	0.9800	C9B—H9BC	0.9800
C10A—C11A	1.520 (8)	C10B—C11B	1.514 (8)
C10A—H10A	0.9900	C10B—H10C	0.9900
C10A—H10B	0.9900	C10B—H10D	0.9900
C11A—C16A	1.382 (11)	C11B—C16B	1.391 (9)
C11A—C12A	1.403 (9)	C11B—C12B	1.396 (11)
C12A—C13A	1.420 (9)	C12B—C13B	1.390 (10)
C13A—C14A	1.412 (11)	C12B—H12B	0.9500
C13A—H13A	0.9500	C13B—C14B	1.413 (10)
C14A—C15A	1.354 (11)	C13B—H13B	0.9500
C14A—H14A	0.9500	C14B—C15B	1.374 (12)
C15A—C16A	1.388 (10)	C14B—H14B	0.9500
C15A—H15A	0.9500	C15B—C16B	1.405 (9)
C16A—H16A	0.9500	C15B—H15B	0.9500
C17A—H17A	0.9800	C17B—H17D	0.9800
C17A—H17B	0.9800	C17B—H17E	0.9800
C17A—H17C	0.9800	C17B—H17F	0.9800
			
C6A—Pd1A—N1A	178.3 (2)	C6B—Pd1B—N1B	178.4 (2)
C6A—Pd1A—Cl2A	88.08 (15)	C6B—Pd1B—Cl2B	89.21 (18)
N1A—Pd1A—Cl2A	90.23 (14)	N1B—Pd1B—Cl2B	89.18 (14)
C6A—Pd1A—Cl1A	90.46 (15)	C6B—Pd1B—Cl1B	91.18 (18)
N1A—Pd1A—Cl1A	91.25 (14)	N1B—Pd1B—Cl1B	90.43 (14)
Cl2A—Pd1A—Cl1A	177.53 (6)	Cl2B—Pd1B—Cl1B	177.21 (5)
C12A—S1A—C17A	102.7 (3)	C16B—S1B—C17B	103.3 (4)
C5A—N1A—C1A	118.4 (6)	C1B—N1B—C5B	117.7 (6)
C5A—N1A—Pd1A	119.4 (3)	C1B—N1B—Pd1B	119.7 (4)
C1A—N1A—Pd1A	122.1 (5)	C5B—N1B—Pd1B	122.6 (5)
C6A—N2A—C8A	111.0 (4)	C6B—N2B—C7B	111.4 (5)
C6A—N2A—C9A	125.1 (5)	C6B—N2B—C9B	125.0 (5)
C8A—N2A—C9A	123.9 (5)	C7B—N2B—C9B	123.5 (6)
C6A—N3A—C7A	109.5 (5)	C6B—N3B—C8B	110.0 (5)
C6A—N3A—C10A	126.6 (5)	C6B—N3B—C10B	125.7 (5)
C7A—N3A—C10A	123.7 (5)	C8B—N3B—C10B	124.1 (5)
N1A—C1A—C2A	122.0 (6)	N1B—C1B—C2B	122.8 (6)
N1A—C1A—H1A	119.0	N1B—C1B—H1B	118.6
C2A—C1A—H1A	119.0	C2B—C1B—H1B	118.6
C3A—C2A—C1A	118.7 (5)	C3B—C2B—C1B	118.3 (7)
C3A—C2A—H2A	120.6	C3B—C2B—H2B	120.9
C1A—C2A—H2A	120.6	C1B—C2B—H2B	120.9
C4A—C3A—C2A	118.9 (7)	C2B—C3B—C4B	119.0 (6)
C4A—C3A—H3A	120.6	C2B—C3B—H3B	120.5
C2A—C3A—H3A	120.6	C4B—C3B—H3B	120.5
C3A—C4A—C5A	120.3 (7)	C5B—C4B—C3B	118.0 (5)
C3A—C4A—H4A	119.9	C5B—C4B—H4B	121.0
C5A—C4A—H4A	119.9	C3B—C4B—H4B	121.0
N1A—C5A—C4A	121.7 (5)	N1B—C5B—C4B	124.1 (7)
N1A—C5A—H5A	119.1	N1B—C5B—H5B	117.9
C4A—C5A—H5A	119.1	C4B—C5B—H5B	117.9
N2A—C6A—N3A	106.4 (5)	N2B—C6B—N3B	105.2 (5)
N2A—C6A—Pd1A	127.1 (4)	N2B—C6B—Pd1B	127.7 (4)
N3A—C6A—Pd1A	126.4 (4)	N3B—C6B—Pd1B	127.0 (5)
C8A—C7A—N3A	107.2 (5)	C8B—C7B—N2B	105.7 (6)
C8A—C7A—H7A	126.4	C8B—C7B—H7B	127.1
N3A—C7A—H7A	126.4	N2B—C7B—H7B	127.1
C7A—C8A—N2A	105.9 (6)	C7B—C8B—N3B	107.6 (5)
C7A—C8A—H8A	127.0	C7B—C8B—H8B	126.2
N2A—C8A—H8A	127.0	N3B—C8B—H8B	126.2
N2A—C9A—H9AA	109.5	N2B—C9B—H9BA	109.5
N2A—C9A—H9AB	109.5	N2B—C9B—H9BB	109.5
H9AA—C9A—H9AB	109.5	H9BA—C9B—H9BB	109.5
N2A—C9A—H9AC	109.5	N2B—C9B—H9BC	109.5
H9AA—C9A—H9AC	109.5	H9BA—C9B—H9BC	109.5
H9AB—C9A—H9AC	109.5	H9BB—C9B—H9BC	109.5
N3A—C10A—C11A	114.5 (4)	N3B—C10B—C11B	114.7 (5)
N3A—C10A—H10A	108.6	N3B—C10B—H10C	108.6
C11A—C10A—H10A	108.6	C11B—C10B—H10C	108.6
N3A—C10A—H10B	108.6	N3B—C10B—H10D	108.6
C11A—C10A—H10B	108.6	C11B—C10B—H10D	108.6
H10A—C10A—H10B	107.6	H10C—C10B—H10D	107.6
C16A—C11A—C12A	119.8 (7)	C16B—C11B—C12B	119.8 (7)
C16A—C11A—C10A	118.9 (7)	C16B—C11B—C10B	122.2 (7)
C12A—C11A—C10A	121.3 (6)	C12B—C11B—C10B	117.9 (7)
C11A—C12A—C13A	118.9 (7)	C13B—C12B—C11B	120.7 (7)
C11A—C12A—S1A	118.3 (5)	C13B—C12B—H12B	119.6
C13A—C12A—S1A	122.8 (5)	C11B—C12B—H12B	119.6
C14A—C13A—C12A	119.2 (7)	C12B—C13B—C14B	119.1 (8)
C14A—C13A—H13A	120.4	C12B—C13B—H13B	120.4
C12A—C13A—H13A	120.4	C14B—C13B—H13B	120.4
C15A—C14A—C13A	120.5 (8)	C15B—C14B—C13B	120.2 (8)
C15A—C14A—H14A	119.7	C15B—C14B—H14B	119.9
C13A—C14A—H14A	119.7	C13B—C14B—H14B	119.9
C14A—C15A—C16A	120.6 (7)	C14B—C15B—C16B	120.4 (8)
C14A—C15A—H15A	119.7	C14B—C15B—H15B	119.8
C16A—C15A—H15A	119.7	C16B—C15B—H15B	119.8
C11A—C16A—C15A	120.9 (8)	C11B—C16B—C15B	119.7 (7)
C11A—C16A—H16A	119.5	C11B—C16B—S1B	118.4 (5)
C15A—C16A—H16A	119.5	C15B—C16B—S1B	121.9 (5)
S1A—C17A—H17A	109.5	S1B—C17B—H17D	109.5
S1A—C17A—H17B	109.5	S1B—C17B—H17E	109.5
H17A—C17A—H17B	109.5	H17D—C17B—H17E	109.5
S1A—C17A—H17C	109.5	S1B—C17B—H17F	109.5
H17A—C17A—H17C	109.5	H17D—C17B—H17F	109.5
H17B—C17A—H17C	109.5	H17E—C17B—H17F	109.5
			
C5A—N1A—C1A—C2A	−0.4 (9)	C5B—N1B—C1B—C2B	−1.8 (9)
Pd1A—N1A—C1A—C2A	−178.0 (5)	Pd1B—N1B—C1B—C2B	−179.8 (5)
N1A—C1A—C2A—C3A	−0.3 (10)	N1B—C1B—C2B—C3B	1.4 (9)
C1A—C2A—C3A—C4A	−0.2 (9)	C1B—C2B—C3B—C4B	−0.5 (9)
C2A—C3A—C4A—C5A	1.4 (9)	C2B—C3B—C4B—C5B	0.1 (9)
C1A—N1A—C5A—C4A	1.6 (8)	C1B—N1B—C5B—C4B	1.4 (8)
Pd1A—N1A—C5A—C4A	179.3 (4)	Pd1B—N1B—C5B—C4B	179.3 (4)
C3A—C4A—C5A—N1A	−2.1 (9)	C3B—C4B—C5B—N1B	−0.6 (9)
C8A—N2A—C6A—N3A	0.5 (6)	C7B—N2B—C6B—N3B	−0.8 (7)
C9A—N2A—C6A—N3A	−176.8 (5)	C9B—N2B—C6B—N3B	177.6 (5)
C8A—N2A—C6A—Pd1A	178.5 (4)	C7B—N2B—C6B—Pd1B	−177.3 (4)
C9A—N2A—C6A—Pd1A	1.2 (8)	C9B—N2B—C6B—Pd1B	1.0 (9)
C7A—N3A—C6A—N2A	0.0 (6)	C8B—N3B—C6B—N2B	−0.1 (6)
C10A—N3A—C6A—N2A	176.2 (5)	C10B—N3B—C6B—N2B	−175.4 (5)
C7A—N3A—C6A—Pd1A	−178.1 (4)	C8B—N3B—C6B—Pd1B	176.5 (4)
C10A—N3A—C6A—Pd1A	−1.8 (8)	C10B—N3B—C6B—Pd1B	1.2 (8)
C6A—N3A—C7A—C8A	−0.5 (6)	C6B—N2B—C7B—C8B	1.3 (7)
C10A—N3A—C7A—C8A	−176.8 (5)	C9B—N2B—C7B—C8B	−177.1 (6)
N3A—C7A—C8A—N2A	0.8 (6)	N2B—C7B—C8B—N3B	−1.3 (7)
C6A—N2A—C8A—C7A	−0.8 (7)	C6B—N3B—C8B—C7B	0.9 (7)
C9A—N2A—C8A—C7A	176.5 (5)	C10B—N3B—C8B—C7B	176.3 (6)
C6A—N3A—C10A—C11A	47.6 (8)	C6B—N3B—C10B—C11B	−50.9 (8)
C7A—N3A—C10A—C11A	−136.7 (6)	C8B—N3B—C10B—C11B	134.4 (6)
N3A—C10A—C11A—C16A	−116.6 (7)	N3B—C10B—C11B—C16B	−62.8 (7)
N3A—C10A—C11A—C12A	65.6 (7)	N3B—C10B—C11B—C12B	121.2 (7)
C16A—C11A—C12A—C13A	0.6 (9)	C16B—C11B—C12B—C13B	0.9 (10)
C10A—C11A—C12A—C13A	178.4 (5)	C10B—C11B—C12B—C13B	176.9 (5)
C16A—C11A—C12A—S1A	−178.6 (5)	C11B—C12B—C13B—C14B	0.2 (9)
C10A—C11A—C12A—S1A	−0.7 (7)	C12B—C13B—C14B—C15B	−0.9 (10)
C17A—S1A—C12A—C11A	156.8 (5)	C13B—C14B—C15B—C16B	0.5 (11)
C17A—S1A—C12A—C13A	−22.4 (6)	C12B—C11B—C16B—C15B	−1.3 (9)
C11A—C12A—C13A—C14A	−0.5 (10)	C10B—C11B—C16B—C15B	−177.2 (5)
S1A—C12A—C13A—C14A	178.7 (6)	C12B—C11B—C16B—S1B	179.0 (5)
C12A—C13A—C14A—C15A	−0.2 (12)	C10B—C11B—C16B—S1B	3.1 (8)
C13A—C14A—C15A—C16A	0.7 (11)	C14B—C15B—C16B—C11B	0.7 (10)
C12A—C11A—C16A—C15A	−0.1 (10)	C14B—C15B—C16B—S1B	−179.6 (6)
C10A—C11A—C16A—C15A	−178.0 (5)	C17B—S1B—C16B—C11B	−156.2 (5)
C14A—C15A—C16A—C11A	−0.5 (10)	C17B—S1B—C16B—C15B	24.1 (6)

### Dichlorido{1-methyl-3-[2-(methylsulfanyl)benzyl]-2H-imidazol-2-ylidene-κC2}(pyridine-κN)palladium(II) (7a) Hydrogen-bond geometry (Å, º)

*Cg*1 is the centroid of the imidazolium ring in molecule *A* and *Cg*4 is the centroid of the imidazolium ring in molecule *B*.

**Table:**

*D*—H···*A*	*D*—H	H···*A*	*D*···*A*	*D*—H···*A*
C2*A*—H2*A*···Cl2*B*	0.95	2.66	3.570 (6)	161
C4*A*—H4*A*···Cl1*A*^i^	0.95	2.83	3.536 (8)	132
C8*A*—H8*A*···Cl2*A*^ii^	0.95	2.77	3.514 (6)	136
C2*B*—H2*B*···Cl1*B*^iii^	0.95	2.78	3.573 (8)	141
C4*B*—H4*B*···Cl2*A*^iv^	0.95	2.72	3.415 (6)	130
C8*B*—H8*B*···Cl1*A*^i^	0.95	2.92	3.710 (6)	142
C9*A*—H9*AB*···*Cg*1^ii^	0.98	2.86	3.745 (6)	150
C9*B*—H9*BB*···*Cg*4^v^	0.98	2.84	3.765 (6)	158

Symmetry codes: (i) *x*−1, *y*, *z*; (ii) −*x*+2, −*y*+2, −*z*+2; (iii) *x*+1, *y*, *z*; (iv) *x*, *y*, *z*−1; (v) −*x*, −*y*+2, −*z*+1.

## References

[bb1] Arduengo, A. J. III, Harlow, R. L. & Kline, M. (1991). *J. Am. Chem. Soc.* **113**, 361–363.

[bb2] Bai, D.-C., Yu, F.-L., Wang, W.-Y., Chen, D., Li, H., Liu, Q. R., Ding, C. H., Chen, B. & Hou, X. L. (2016). *Nat. Commun.* **7**, 11806. 10.1038/ncomms11806 PMC4906412 27283477

[bb3] Bärnighausen, H. (1980). *MATCH **9*** 139–175.

[bb4] Bruker (2020). *APEX3*, *SAINT* and *TWINABS*. Bruker AXS Inc., Madison, Wisconsin, USA.

[bb5] Bruno, I. J., Cole, J. C., Edgington, P. R., Kessler, M., Macrae, C. F., McCabe, P., Pearson, J. & Taylor, R. (2002). *Acta Cryst.* B**58**, 389–397. 10.1107/s0108768102003324 12037360

[bb6] Çekirdek, S., Yaşar, S. & Özdemir, İ. (2014). *Appl. Organomet. Chem.* **28**, 423–431.

[bb7] Fortman, G. C. & Nolan, S. P. (2011). *Chem. Soc. Rev.* **40**, 5151–5169. 10.1039/c1cs15088j 21731956

[bb8] Groom, C. R., Bruno, I. J., Lightfoot, M. P. & Ward, S. C. (2016). *Acta Cryst.* B**72**, 171–179. 10.1107/S2052520616003954 PMC4822653 27048719

[bb9] Huynh, H. V., Yeo, C. H. & Chew, Y. X. (2010). *Organometallics* **29**, 1479–1486.

[bb10] Kantchev, E., O’Brien, C. & Organ, M. (2007). *Angew. Chem. Int. Ed.* **46**, 2768–2813. 10.1002/anie.200601663 17410611

[bb11] Karthik, S. & Gandhi, T. (2018). *New J. Chem.* **42**, 15811–15819.

[bb12] Krause, L., Herbst-Irmer, R., Sheldrick, G. M. & Stalke, D. (2015). *J. Appl. Cryst.* **48**, 3–10. 10.1107/S1600576714022985 PMC4453166 26089746

[bb13] Lohre, C., Fröhlich, R. & Glorius, F. (2008). *Synthesis* pp. 2221–2228.

[bb14] Macrae, C. F., Sovago, I., Cottrell, S. J., Galek, P. T. A., McCabe, P., Pidcock, E., Platings, M., Shields, G. P., Stevens, J. S., Towler, M. & Wood, P. A. (2020). *J. Appl. Cryst.* **53**, 226–235. 10.1107/S1600576719014092 PMC6998782 32047413

[bb15] Müller, U. & de la Flor, G. (2024). *Symmetry Relationships Between Crystal Structures* 2nd ed. International Union of Crystallography Texts on Crystallography. Oxford University Press.

[bb16] O’Brien, C. J., Kantchev, E. A. B., Valente, C., Hadei, N., Chass, G. A., Lough, A., Hopkinson, A. C. & Organ, M. G. (2006). *Chem. Eur. J.* **12**, 4743–4748. 10.1002/chem.200600251 16568494

[bb17] Pasyukov, D. V., Shevchenko, M. A., Astakhov, A. V., Minyaev, M. E., Zhang, Y., Chernyshev, V. M. & Ananikov, V. P. (2023). *Dalton Trans.* **52**, 12067–12086. 10.1039/d3dt02296j 37581341

[bb18] Pasyukov, D. V., Shevchenko, M. A., Shepelenko, K. E., Khazipov, O. V., Burykina, J. V., Gordeev, E. G., Minyaev, M. E., Chernyshev, V. M. & Ananikov, V. P. (2022). *Angew. Chem. Int. Ed.* **61**, e202116131. 10.1002/anie.202116131 34963027

[bb19] Sheldrick, G. M. (2015*a*). *Acta Cryst.* A**71**, 3–8.

[bb20] Sheldrick, G. M. (2015*b*). *Acta Cryst.* C**71**, 3–8.

[bb21] Shevchenko, M. A., Pasyukov, D. V., Minyaev, M. E. & Chernyshev, V. M. (2024). *Russ. Chem. Bull.* **73**, 917–931.

[bb22] Spek, A. L. (2020). *Acta Cryst.* E**76**, 1–11. 10.1107/S2056989019016244 PMC6944088 31921444

[bb23] Westrip, S. P. (2010). *J. Appl. Cryst.* **43**, 920–925.

[bb24] Yang, L., Powell, D. R. & Houser, R. P. (2007). *Dalton Trans.* pp. 955–964. 10.1039/b617136b 17308676

[bb25] Yue, C., Xing, Q., Sun, P., Zhao, Z., Lv, H. & Li, F. (2021). *Nat. Commun.* **12**, 1875. 10.1038/s41467-021-22084-5 PMC7994585 33767184

